# Pharmacologic treatment of fibromyalgia: an update

**DOI:** 10.3389/fphar.2025.1651181

**Published:** 2025-10-10

**Authors:** Yang Li, Yunshen Li, Shiya Wu, Meijuan Long, Xinyi Chen, Yidan Wang, Jianan Zhao, Bingheng He, Cen Chang, Juan Jiao

**Affiliations:** ^1^ Rheumatology Department, Guang’anmen Hospital, China Academy of Chinese Medical Sciences, Beijing, China; ^2^ Guanghua Hospital Affiliated to Shanghai University of Traditional Chinese Medicine, Shanghai University of Traditional Chinese Medicine, Shanghai, China; ^3^ Baoding Hospital affiliated to Guang 'anmen Hospital, Beijing, Hebei, China; ^4^ Tongren Hospital Shanghai Jiao Tong University School Of Medicine, Shanghai, China; ^5^ Institute of Arthritis Research in Integrative Medicine, Shanghai Academy of Traditional Chinese Medicine, Shanghai, China

**Keywords:** fibromyalgia, pharmacological treatment, pregabalin, duloxetine, drug development

## Abstract

Fibromyalgia (FM) is a chronic pain disorder characterized by widespread musculoskeletal pain, frequently accompanied by fatigue, sleep disturbances, cognitive dysfunction, and mood disorders. Although the etiology is multifactorial, involving genetic, hormonal, immunological, and environmental contributors, the exact pathophysiological mechanisms remain unclear, and the heterogeneity of symptoms poses significant challenges for diagnosis and management. Diagnostic criteria have evolved over the years, with newer approaches reducing some limitations, yet sex-related differences in clinical presentation continue to challenge timely and accurate diagnosis. Pharmacological therapy remains unsatisfactory: only pregabalin, duloxetine, and milnacipran have gained U.S. FDA approval, providing clinically meaningful relief in a minority of patients, and their frequent adverse events limit adherence. Numerous other agents, including cyclobenzaprine, gabapentinoids, NMDA antagonists, cannabinoids, and sodium oxybate, have been evaluated, but most remain investigational or limited by safety concerns. Current guidelines emphasize that pharmacological interventions alone are insufficient and should be integrated with non-pharmacological strategies, especially patient education, exercise, and psychological support. This review summarizes the evidence on available and emerging pharmacological options for FM, highlights their limitations, and underscores the need for individualized, multimodal treatment strategies to improve patient outcomes and quality of life.

## 1 Introduction

Fibromyalgia (FM) is characterized primarily by chronic widespread musculoskeletal pain and is often accompanied by symptoms such as sleep disturbances, fatigue, and reduced physical function. It also leads to “fibro fog,” which includes various mental and cognitive abnormalities such as anxiety, depression, memory loss, and diminished attention (Clauw, 2014). Current prevalence rates range from 0.7% to 9.3%, with the global average estimated at approximately 2.7% (Sarzi-Puttini et al., 2020). The etiology and pathophysiological mechanisms of FM remain unclear, though it is widely regarded as a pain regulation disorder, classified as a central sensitization syndrome (Giorgi et al., 2023). About 50% of patients with FM struggle to maintain normal work, and more than one-fifth become unemployed within 10 months of diagnosis (Schaefer et al., 2016). The associated medical costs are substantial, with U.S. Medicare data showing that the annual healthcare expenditure per patient can reach up to $35,920 (D’Onghia et al., 2022), marking FM as a chronic disease with a significant societal burden.

In addition, sex-related biological and symptom-expression differences can influence how readily patients meet certain diagnostic frameworks. Large epidemiologic surveys report higher pain prevalence in women than in men (Fillingim et al., 2009). Women, on average, exhibit lower pain thresholds—potentially related to fluctuations in estrogen—whereas higher testosterone levels in men are thought to exert antinociceptive effects (Bartley and Fillingim, 2013; Lenert et al., 2021). Under the 1990 ACR criteria, which required ≥11/18 tender points, these differences likely increased the probability of diagnosis in women relative to men (Wolfe et al., 1990; Moshrif et al., 2022). Application of the 2016 ACR revisions (without tender-point examination) has narrowed—but not eliminated—the sex gap; moreover, in clinical practice fibromyalgia in men may still be under-recognized (Liu et al., 2025).

Due to the ongoing lack of effective pharmacological treatments for FM, only three medications have been approved by the U.S. Food and Drug Administration thus far: the anticonvulsant pregabalin and the antidepressants duloxetine and milnacipran, which are used in many countries and regions worldwide. However, their clinical efficacy remains unsatisfactory. These drugs are strongly recommended only in FM guidelines from Canada (Fitzcharles et al., 2012), Israel (Ablin et al., 2013), and Italy (Ariani et al., 2021), while Germany provides a weak recommendation (Häuser et al., 2010), and the European Alliance of Associations for Rheumatology recommends their use only in patients with severe pain or sleep disturbances (Macfarlane et al., 2017). In China, these drugs are recommended only for patients with inadequate responses to non-pharmacological interventions (China Association of Chinese Medicine Rheumatology Society, Fibromyalgia Research Group of Traditional Chinese and Western Medicine in Rheumatology and Immunology, Capital Institute of Rheumatology and Immunology of Integrated Chinese and Western Medicine, 2023). The limited therapeutic benefits of these medications, coupled with their frequent adverse effects, contribute to their limited endorsement. Systematic reviews indicate that pregabalin (Üçeyler et al., 2017), duloxetine (Rodrigues-Amorim et al., 2020), and milnacipran (Häuser et al., 2013) provide significant relief for approximately 30% of FM-related pain, but their efficacy for symptoms such as fatigue, sleep disturbances, and impaired physical function is limited. Additionally, these drugs are associated with a high incidence of adverse reactions, with common side effects of pregabalin including edema, drowsiness, dizziness, and weight gain, with an overall incidence rate of 85%–90% (Pauer et al., 2011). Due to the poor tolerability of these side effects, adherence rates for pregabalin, milnacipran, and duloxetine are only 47%, 43%, and 59%, respectively, with discontinuation rates as high as 64.6%, 72.1%, and 51.5% within 1 year (Liu et al., 2016). It has been over a decade since the approval of the latest drug, milnacipran, and despite ongoing efforts by pharmaceutical companies worldwide, no new drugs have been approved. As a result, only a minority of patients derive substantial benefit, while the majority face the clinical challenge of inadequate efficacy or poor tolerability, ultimately leading to treatment discontinuation.

Although current pharmacotherapeutic regimens for FM are not ideal, and clinical guidelines mostly recommend that patients prioritize non-pharmacological therapies such as exercise and patient education (Macfarlane et al., 2017; Antunes et al., 2023) for treatment, over 90% of FM patients still seek medication-based treatment (Siracusa et al., 2021). The aim of this review is to summarize all drug development and application efforts for FM to date, identify medications that warrant further investigation, and highlight the challenges and issues in current drug research. This review seeks to provide valuable insights and guidance for future pharmacological research in the treatment of FM.

## 2 Literature search methodology

This review includes a search of all drug-related therapeutic clinical trials, case reports, and mechanistic studies involving FM patients or animal models, available on PubMed and Web of Science as of July 2024. The search encompassed studies written in English, Spanish, Italian, or French. Given the large number of retrieved clinical studies for some drugs, we prioritized high-quality systematic reviews when summarizing the findings (Table 1).

**TABLE 1:** Summary of progress in FM therapeutic drug development

Drugs	Research progress	Mechanism	Symptoms that can be improved	Advantages of efficacy	Limitations of efficacy	Major adverse events	Incidence of adverse effects
Milnacipran (Cording et al., 2015; Gupta et al., 2021)	FDA-approved	Serotonin-norepinephrine reuptake inhibitor (SNRI); Inhibits presynaptic reuptake of serotonin (5-hydroxytryptamine, 5-HT) and norepinephrine (NE) to enhance central pain regulation	Pain, fatigue, physical function, sleep quality, mood, quality of life	Specializing in the treatment of fatigue	A small number of patients benefit; increases the risk of suicide in young patients	Nausea, headache, constipation, dizziness, insomnia	86% (vs. 78% placebo)
Duloxetine (Lian et al., 2020; Migliorini et al., 2023; Arnold et al., 2011; Arnold et al., 2010a; Mohs et al., 2012; Lunn et al., 2014; Chappell et al., 2009; Arnold et al., 2005)	FDA-approved	SNRI; inhibits presynaptic reuptake of 5-HT and NE to enhance central pain regulation	Pain, global disease impact (PGIC), fatigue, anxiety, depression	Specializing in the treatment of pain	A small number of patients benefit	Nausea, constipation, hyperhidrosis, headache, dry mouthetc.	82.6% (vs. 69.7% placebo)
Pregabalin (Taylor et al., 2007; Derry et al., 2016; Arnold et al., 2018; Arnold et al., 2015; Migliorini et al., 2022)	FDA-approved	Gamma-aminobutyric acid (GABA) analogue; binds to calcium channel α2δ subunit, reducing calcium influx and excitatory neurotransmitter release	Pain, fatigue, sleep quality and PGIC	Its efficacy is not affected by demographic characteristics	A small number of patients benefit	Dizziness, somnolence, weight gain, dry mouth, nausea, blurred visionetc.	70%–90%
Cyclobenzaprine (Moldofsky et al., 2011; Lederman et al., 2023; Carette et al., 1994; Santandrea et al., 1993; Cantini et al., 1994; Fossaluzza and De Vita, 1992)	Phase III clinical trials	Centrally acting muscle relaxant; antagonizes 5-HT2A, ADRA2, H1 receptors; reduces motor neuron excitability	Pain, fatigue, depression level, sleep, irritable bowel syndrome and stiffness	Sublingual formulation has good tolerability; similar efficacy to amitriptyline (short-term)	Long-term effects (6 months) similar to placebo; higher doses (30 mg/d) increase side effects	Oral hypoesthesia, oral paresthesia, abnormal taste, etc.	27% (10 mg/d) - 84% (30 mg/d)
Sodium oxybate (Scharf et al., 2003; Russell et al., 2009; Russell et al., 2011; Spaeth et al., 2012; Spaeth et al., 2013)	Phase III clinical trials	GABA-B receptor agonist; sedative effects; mechanism not fully clarified	Pain, sleep disturbances, fatigue, physical function, quality of life	Long-term efficacy (38 weeks) and tolerability; significant sleep/pain improvement	Abuse potential (structurally similar to GHB)	Headache, nausea, dizziness, vomiting, diarrhea, anxiety, sinusitis	46.7%–48.4% (vs. 29.0% placebo)
NYX-2925 (Ghoreishi-Haack et al., 2018; Houck et al., 2019)	Phase II clinical trials	Novel N-methyl-D-aspartate (NMDA) receptor modulator; regulates NMDA activity	Neuropathic pain (animal models)	Unclear	Unclear	Unclear	Unclear
Ferric carboxymaltose (Ortanci et al., 2010; Hamarat et al., 2023; Boomershine et al., 2018)	Phase II clinical trials	Intravenous iron supplement; replenishes iron stores; iron is a cofactor for 5-HT/NE synthesis	Pain, fatigue, global disease impact	Fewer adverse effects	Only effective in iron-deficient patients	Flushing, nausea, and dizziness	29.3% (vs. 5% placebo)
Mirtazapine (Banerjee et al., 2021; Neyama et al., 2020; Yeephu et al., 2013; Miki et al., 2016; Mehta et al., 2022)	Phase IIa clinical trials	Enhances central NE/5-HT activity; antagonizes H1 receptors for sedation	Pain, quality of life, sleep quality and overall disease impact	Improves sleep independently of pain relief	Less effective than duloxetine (46.7% vs. 89.4% symptom improvement); limited study quantity	Drowsiness, weight gain, increased appetite	68.8% (vs. 56.7% placebo)
ASP0819 (Arnold et al., 2020; Takeshita et al., 2021)	Phase IIa clinical trials	Non-opioid KCa 3.1 channel opener; reverses abnormal sensory neuron discharges; modulates peripheral nociceptive signaling	Pain, sleep quality, global disease impact	Unclear	Unclear	Headache, joint pain, nausea and vomiting	68%
Testosterone Gel (White et al., 2015)	Phase I/II pilot trial	Topical androgen; regulates pain thresholds; modulates neuroendocrine/pain signaling pathways	Muscle pain, stiffness, fatigue, tender point count	No adverse effects on cardiovascular/hepatic/renal function; no treatment-related adverse events	Limited to small female cohort (n = 12); no large-scale or long-term trials	None reported	0%
Amitriptyline (Häuser et al., 2011; Farag et al., 2022; Moore et al., 2019)	10+ clinical studies; systematic reviews with meta-analyses	Tricyclic antidepressant (SNRI); inhibits 5-HT/NE reuptake; modulates H1 receptors	Pain, sleep quality, fatigue, quality of life	Greatest sleep and fatigue improvement vs. other drugs; similar acceptability to placebo	Studies have small samples, design flaws; efficacy for tender points controversial	Dry mouth, weight gain, lethargy, dizziness, etc.	78% (vs. 47% placebo)
Naltrexone Hydrochloride (Younger and Mackey, 2009; Younger et al., 2009; Younger et al., 2013; Parkitny and Younger, 2017; Jackson et al., 2021; Bested et al., 2023; Due Bruun et al., 2024).	7 clinical trials	Blocks μ-opioid receptors; low doses reduce pro-inflammatory cytokines (IL-1β, TNF-α)	Pain, memory, life satisfaction	Early trials showed anti-inflammatory effects; improves memory related to FM pain	Recent high-quality trials show no clinically significant pain relief; efficacy controversial	Insomnia, vivid dreams	Not reported in recent trials; 100% in small retrospective cohort
Palmitoylethanolamide (Gatti et al., 2012; Luongo et al., 2013; Schweiger et al., 2019; Del Giorno et al., 2015; Salaffi et al., 2023)	4 clinical trials	Endocannabinoid-like substance; interacts with CB1/CB2	Unclear	It can enhance the efficacy of pregabalin combined with duloxetine	Unclear	Diarrhea, dyspepsia, bloating, constipation, vomiting	10.0%
Venlafaxine (Kleinman et al., 2011; VanderWeide et al., 2015; Dwight et al., 1998; Díaz-Marsá et al., 2011)	3 clinical trials	5-HT or NE modulators	Pain, anxiety, depression, global impact of illness, PGIC.	It is more economical and may have better efficacy for FM complicated with mental diseases	Unclear	Dry mouth, weight gain, somnolence, dizziness, fatigue, constipation, etc.	77% (vs. 47% placebo)
Esreboxetine (Arnold et al., 2012; Arnold et al., 2010b)	2 RCTS	Selective norepinephrine reuptake inhibitor (NARI); inhibits NE reuptake	Pain, fatigue, quality of life, PGIC	Unclear	Unclear	Insomnia, constipation, dry mouth, nausea, dizzinessetc.	57.7%–71.6% (vs. 30.7%–57.1% placebo)
Gabapentin (Arnold et al., 2007; North et al., 2016)	2 clinical trials	GABA analogue; regulates calcium channels, reducing glutamate/NE/substance P release	Pain, fatigue, quality of life, PGIC	Extended-release formulation improves bioavailability, reduces side effects	Not effective for depression	Drowsiness, dizziness, irritability, weight gain, dry eyes, difficulty concentrating, etc.	79.30%
Memantine hydrochloride (Fayed et al., 2014; Olivan-Blázquez et al., 2014)	2 RCTS	NMDA receptor antagonist; modulates glutamate signaling, protects neurons	Pain, overall impact of disease, anxiety, quality of life, PGIC	Improves multiple symptoms (pain, mood, cognition); good tolerability	Unclear	Dizziness, headache	96.70%
Paroxetine (Patkar et al., 2007; Sencan et al., 2004)	1 RCT; 1 clinical trial	Selective serotonin reuptake inhibitor (SSRI); inhibits 5-HT reuptake	PGIC	Similar efficacy to aerobic exercise for depression; good tolerability	No significant pain/VAS improvement vs. placebo	Somnolence, dry mouth, female reproductive organ disorders, ejaculation disorders, impotence, etc.	65.5% (vs. 58.6% placebo)
Ketamine (Graven-Nielsen et al., 2000)	1 clinical trial; case selection based on pain response	NMDA receptor antagonist; inhibits NMDA channel activity, regulating neural signaling	Pain	Rapid analgesic effect in responsive patients	Unclear	Unclear	Unclear
Interferon-alpha (Russell et al., 1999a; Russell et al., 1999b)	2 small trials	Antiviral; modulates immune function (reduces CD4^+^ T cell subsets)	Morning stiffness, physical function	Favorable safety profile	Only 50 IU dose effective; no improvement in pain/fatigue; limited evidence	Unclear	Unclear
Alpha-1 Antitrypsin (AAT) (Blanco et al., 2004; Alegre et al., 2012)	1 case report; 1 clinical trial	Serine protease inhibitor	Unclear	Symptom relief in AAT-deficient FM patients (case report)	No significant improvement in randomized placebo-controlled trial	Unclear	Unclear
Dehydroepiandrosterone (DHEA) (Finckh et al., 2005)	1 double-blind crossover trial	Hormone precursor	None	Increases serum DHEA sulfate levels	No clinical benefit for FM symptoms; androgen-related side effects	Oily skin, acne, increased body hair	Unclear
Tetrahydrocannabinol (Hindley et al., 2020; Boehnke et al., 2021; Schley et al., 2006; Weber et al., 2009)	1 clinical trial; 1 multicenter survey	Psychoactive cannabinoid; binds to CB1 receptors; modulates central pain signaling	Pain (significant reduction in intensity), mental health	Higher THC doses (10–15 mg) relieve electrically induced pain; improves psychological measures	High discontinuation rate; abuse potential; cognitive impairment	Fatigue, dizziness, mental confusion, nausea/vomiting, restlessness	Unclear
Mirogabalin (Vinik et al., 2014; Baba et al., 2019; Murasawa et al., 2020; Murasawa et al., 2021; Saeki et al., 2019; Arnold et al., 2019)	1 animal experiment; 1 negative human trial	Calcium channel inhibitor; high selectivity for α2δ-1 subunit (slower dissociation than pregabalin)	Pain (animal models: reduced hyperalgesia), anxiety-like behavior, cognitive impairment (rats)	Potential long-lasting analgesic effects (animal data)	No significant pain relief in human trials	Unclear	Unclear
Tramadol (Biasi et al., 1998)	1 clinical trial	Binds to μ-opioid receptors; inhibits NE/5-HT reuptake	Pain	Unclear	High overdose/misuse risk	Not reported in FM trial (known effects: nausea, constipation, dizziness in other indications)	Unclear
Flupirtine (Stoll, 2000)	2 case reports	Unclear	Pain, sleep disturbances, fatigue, and depressive symptoms	Unclear	Unclear	Unclear	Unclear
Cannabidiol (CBD) (Hindley et al., 2020; Boehnke et al., 2021)	Online surveys	Non-psychoactive cannabinoid; interacts with CB1/CB2 receptors	Pain, fatigue, anxiety, cognition (self-reported in surveys)	Non-addictive; no psychoactive effects; potential substitute for opioids/GABA analogs	Unclear	Unclear	Unclear
ASP8062 (Murai et al., 2019)	1 animal experiment	Novel GABA-B receptor positive allosteric modulator; enhances GABA-B activity	Pain (rat FM models); sleep electroencephalogram activity	Unclear	Unclear	Unclear	Unclear
Neurotropin (Nasu et al., 2019)	1 animal experiment	Unclear	Depressive-like behavior (rat models); FM pain (investigational)	Unclear	Unclear	Unclear	Unclear

## 3 Advances in drug research targeting the two major mechanisms of FM

### 3.1 Drugs modulating serotonin or norepinephrine

#### 3.1.1 Duloxetine

Duloxetine, a serotonin and norepinephrine reuptake inhibitor, is one of the drugs approved by the U.S. Food and Drug Administration (FDA) for the treatment of FM. A 2020 systematic review (Lian et al., 2020) included seven studies involving a total of 2,642 participants, all of which were double-blind, parallel-group, placebo-controlled trials. The results showed that duloxetine (30–120 mg/d) had a significant effect on pain relief compared to placebo (SMD -0.26; 95% CI -0.37 to −0.16). The risk ratio for achieving 30% pain relief was 1.31 (95% CI 1.19–1.44), and the risk ratio for achieving 50% pain relief was 1.46 (95% CI 1.28–1.67). There was also a significant improvement in Patient Global Impression of Change (PGIC) (SMD -0.28; 95% CI -0.36 to −0.20). Adverse events were more common in the duloxetine group compared to the placebo group (RR 1.17, 95% CI 1.12–1.23). The incidence of experiencing at least one adverse event was significantly higher in the duloxetine group at 82.6%, compared to 69.7% in the placebo group. Common adverse events included nausea, constipation, hyperhidrosis, headache, dry mouth, drowsiness, and insomnia. A 2023 review involving 3,432 patients from 11 randomized controlled trials (RCT) showed that duloxetine was superior to placebo in improving Fibromyalgia Impact Questionnaire (FIQ) scores, PGIC, and pain relief (Migliorini et al., 2023). Although there were no systematic reviews analyzing other FM symptoms, an RCT demonstrated that 60–120 mg/d of duloxetine significantly improved patients’ Multidimensional Fatigue Inventory scores, as well as depression and anxiety scores, after 12 weeks of treatment (Arnold et al., 2011). In another randomized, double-blind, placebo-controlled trial, duloxetine at 60–120 mg/d significantly reduced the total score of the Beck Depression Inventory (BDI) and improved the mental health score of the SF-36 after 12 weeks of treatment in FM patients (Arnold et al., 2010a). However, some studies have found that duloxetine does not improve cognitive function (Mohs et al., 2012). The optimal dosage of duloxetine remains controversial. Systematic reviews and meta-analyses have found that doses above 30 mg/d are necessary to achieve therapeutic effects (Lunn et al., 2014). Some studies have shown that 120 mg/d of duloxetine provides no significant difference in pain relief compared to the 60 mg/d dose, and may even be less effective (Arnold et al., 2010a; Chappell et al., 2009; Arnold et al., 2005). Additionally, other studies have found no difference in pain improvement between the 30–60 mg/d doses and placebo (Migliorini et al., 2023). These findings suggest that the dosage of duloxetine in clinical practice should be flexibly adjusted based on the patient’s response.

Despite its proven clinical efficacy, the benefits of duloxetine remain limited. Studies have shown that the numbers needed to treat for an additional beneficial outcome (NNTB) with 60 mg/d duloxetine to achieve a 50% or greater reduction in pain for FM patients is approximately 8 (Lunn et al., 2014), meaning that only one in every eight patients treated can be expected to experience at least a 50% reduction in pain. This indicates that only a small portion of patients derive significant benefit from duloxetine treatment.

#### 3.1.2 Milnacipran

Milnacipran, another serotonin and norepinephrine reuptake inhibitor, is approved for the treatment of FM in a few countries, such as Canada and the United States. A Cochrane systematic review (Cording et al., 2015) (6 RCTs, n = 4,238) showed that milnacipran at doses of 100 mg or 200 mg provided at least a 30% reduction in pain for approximately 40% of participants. The incidence of adverse events (86%) was similar to that of the placebo group (78%), but the 200 mg dose was more likely than the 100 mg dose to lead to treatment discontinuation due to adverse events. A 2021 review (Gupta et al., 2021) of 37 studies on milnacipran for FM revealed that it demonstrated good efficacy and tolerability in multiple RCTs, improving pain, fatigue, physical function, sleep quality, mood, and quality of life, with effects lasting up to 3 years. Compared to other drugs like amitriptyline, duloxetine, and pregabalin, milnacipran was more effective in alleviating fatigue. Common side effects include nausea and headaches, but there is an increased risk of suicide in children, adolescents, and young adults, which has resulted in a black box warning, making it unsuitable for younger patients.

Overall, milnacipran can improve multiple symptoms of FM with good tolerability and lasting effects, but it still has the limitation that only a small proportion of patients benefit significantly (Cording et al., 2015).

#### 3.1.3 Amitriptyline

Amitriptyline, also a serotonin and norepinephrine reuptake inhibitor, was evaluated in a 2011 systematic review (Häuser et al., 2011) (n = 6,152, 19 clinical studies) comparing amitriptyline, duloxetine, and milnacipran for the treatment of FM. The results showed that FM patients treated with amitriptyline experienced a 30% reduction in pain (RR 1.60; 95% CI 1.15–2.24), moderate efficacy in improving sleep quality (SMD -0.56; 95% CI -0.78 to −0.34), and mild efficacy in reducing fatigue (SMD -0.44; 95% CI -0.71 to −0.16).A 2022 systematic review (Farag et al., 2022) comparing the efficacy and acceptability of amitriptyline, pregabalin, duloxetine, and milnacipran in alleviating FM symptoms found that, compared to placebo, 120 mg duloxetine had the best effect on pain relief (SMD -0.33; 95% CI -0.36 to −0.30). Amitriptyline showed the greatest improvement in sleep (SMD -0.97; 95% CI -1.10 to −0.83), fatigue (SMD -0.64; 95% CI -0.75 to −0.53), and quality of life (SMD -0.80; 95% CI -0.94 to −0.65). Moreover, the acceptability of amitriptyline was similar to that of placebo. However, another systematic review (Moore et al., 2019) found that amitriptyline performed better in achieving at least 50% pain relief (RR 3.0; 95% CI 1.7–4.9), but its efficacy in alleviating fatigue, improving sleep, quality of life, or reducing tender points remains controversial. The incidence of adverse events (78%) was higher in the amitriptyline group compared to the placebo group (47%).

As noted in systematic review analyses, studies on the use of amitriptyline for FM treatment generally suffer from small sample sizes, design flaws, and selective reporting, leading to overall low research quality. While the available data indicate potential benefits in treating FM, high-quality clinical trials are still needed to confirm its efficacy.

#### 3.1.4 Mirtazapine

Mirtazapine enhances norepinephrine and serotonin activity in the central nervous system, and its antagonism of H1 receptors can produce sedative effects and promote sleep (Banerjee et al., 2021). In animal studies, intraperitoneal injection of 1 mg/kg or intracerebroventricular injection of 1 μg mirtazapine reduced hyperalgesia in a mouse model of FM-like pain induced by intermittent cold stress (Neyama et al., 2020). A 2004 open-label trial (Yeephu et al., 2013) (n = 29) found that 6 weeks of mirtazapine treatment (dose unspecified) alleviated pain, sleep disturbances, and fatigue. In a 2013 randomized controlled trial, pain improvement with 15 mg/d and 30 mg/d of mirtazapine was similar to the placebo group, but significant improvements were seen in the Jenkins Sleep Scale and FIQ scores, with overall good tolerability (Yeephu et al., 2013). In a 2016 Phase IIa trial in Japan (Miki et al., 2016), mirtazapine at 15 mg/d for 1 week followed by 30 mg/d for 11 weeks resulted in significant pain score reductions compared to placebo from week six, with 45.5% of intervention patients (n = 211) achieving a ≥30% reduction in pain at the study’s endpoint. Mirtazapine also improved pain-related quality of life, including the Short-Form 36 Questionnaire score and the Japanese version of the FIQ score. Adverse events were slightly higher in the mirtazapine group compared to placebo (68.8% vs. 56.7%), mainly including drowsiness (32.1% vs. 7.4%), weight gain (17.7% vs. 0.9%), and increased appetite (11.6% vs. 3.3%). In a recent retrospective study (Mehta et al., 2022) comparing the efficacy of mirtazapine and duloxetine (n = 158), the proportion of patients showing symptom improvement was lower in the mirtazapine group (46.7%) compared to the duloxetine group (89.4%), while the incidence of adverse events was higher in the mirtazapine group (100% vs. 77%).

Although mirtazapine has shown some efficacy, its effectiveness and safety appear to be inferior to duloxetine. Currently, the number of studies on mirtazapine is still limited, and more high-quality research is needed to confirm its role in FM treatment.

#### 3.1.5 Venlafaxine

Venlafaxine, a serotonin and norepinephrine reuptake inhibitor, has been studied less frequently for FM treatment, but its prescription rate can reach up to 22.5% in specific patient groups (e.g., employees), suggesting that many patients are using this medication (Kleinman et al., 2011). A systematic review indicated that venlafaxine has a generally favorable effect on pain-related outcomes. Although the supporting evidence is limited, venlafaxine is generally well-tolerated and more cost-effective compared to other SNRIs (VanderWeide et al., 2015).

The response of FM patients to venlafaxine treatment may be related to coexisting psychological disorders. A small open-label trial found that venlafaxine at doses of 37.5 mg/d to 300 mg/d significantly reduced FM patients’ McGill Pain Questionnaire scores, Hamilton Depression scores, and Hamilton Anxiety scores after 8 weeks of treatment. Depression and anxiety were predictors of a better response to venlafaxine (Dwight et al., 1998). Díaz-Marsá et al. found that after 6 months of venlafaxine treatment at flexible doses (150 mg/d to 300 mg/d), FM patients showed significant reductions in PGIC scores and FIQ scores, with the reduction in FIQ scores being significantly greater in patients with depression compared to those without depression (Díaz-Marsá et al., 2011). However, another small open-label trial reached conflicting conclusions, showing that 75 mg/d of venlafaxine for 12 weeks significantly reduced average pain intensity and disability scores in FM patients, but these improvements were independent of venlafaxine’s effects on depression and anxiety scores (Sayar et al., 2003). The incidence of adverse events (Díaz-Marsá et al., 2011) was approximately 77% (placebo group 47%), with common side effects including dry mouth, weight gain, drowsiness, dizziness, fatigue, and constipation.

Overall, most clinical studies on venlafaxine for FM are open-label, and the related evidence is relatively limited. More randomized, placebo-controlled trials are needed to confirm its therapeutic effects in FM treatment.

#### 3.1.6 Paroxetine

Paroxetine, a first-generation selective serotonin reuptake inhibitor, is primarily used to treat conditions such as depression and anxiety, and it has shown some efficacy in the treatment of FM. In a 12-week randomized, double-blind, placebo-controlled trial (Patkar et al., 2007) (n = 112), extended-release paroxetine (12.5–62.5 mg/d) significantly reduced the FIQ total score by ≥ 25% in FM patients compared to the placebo group. However, improvements in pain visual analogue scale (VAS) scores and overall disease impression were similar to those of the placebo. The overall tolerability was good, with 65.5% of patients reporting at least one adverse event, a higher rate than the placebo group (58.6%). Common adverse effects included drowsiness and dry mouth. In another study (Sencan et al., 2004) comparing the effects of paroxetine and aerobic exercise on FM treatment, both paroxetine (20 mg/d) and aerobic exercise (3 times per week, 40 min per session) significantly reduced pain VAS and BDI scores after 6 weeks, with similar efficacy between the two treatments. More evidence is still needed to fully support the efficacy of paroxetine in FM treatment.

#### 3.1.7 Esreboxetine

Esreboxetine, a selective norepinephrine reuptake inhibitor, has shown potential in relieving FM symptoms based on recent high-quality RCTs. Esreboxetine at doses of 4 mg/d, 8 mg/d, and 10 mg/d over 14 weeks significantly improved pain scores, FIQ scores, and PGIC scores compared to placebo, though there was no significant difference in SF-36 physical function scores between the groups (Arnold et al., 2012). The incidence of adverse events was 57.7%, higher than the placebo group (30.7%). In another multicenter, randomized, placebo-controlled trial, flexible doses of esreboxetine (2 mg/d-8 mg/d) significantly reduced the average weekly pain score by week 8 compared to placebo, with a higher proportion of patients achieving a ≥30% reduction in pain. Significant improvements were also observed in FIQ total scores, including fatigue and quality of life. Adverse events were more frequent in the esreboxetine group (71.6% vs. 57.1%), with the most common being constipation (17.2% vs. 5.3%), insomnia (15.7% vs. 3.0%), dry mouth (15.7% vs. 2.3%), and headache (10.4% vs. 2.3%) (Arnold et al., 2010b).

### 3.2 Drugs affecting the GABA system

#### 3.2.1 Pregabalin

Pregabalin is a γ-aminobutyric acid (GABA) analogue that exerts its antiepileptic, anxiolytic, and analgesic effects by binding to the α2δ subunit of calcium channels, thereby reducing calcium ion influx (Taylor et al., 2007). It is also one of the FDA-approved medications for the treatment of FM.

A 2016 Cochrane systematic review (Derry et al., 2016), which included five high-quality RCTs involving 3,283 participants, found that pregabalin (150 mg–600 mg/d) was superior to placebo in alleviating pain symptoms and improving PGIC scores. Approximately 27% of patients experienced at least a 30% reduction in pain, with the 450 mg dose showing the most effective pain relief (RR 1.64; 95% CI 1.28–2.10). A 2018 literature review summarized 37 studies on the use of pregabalin in FM treatment, showing that pregabalin significantly improved pain, sleep quality, and overall patient condition. The safety and tolerability of pregabalin were consistent across all studies, and its efficacy was not influenced by demographic or clinical characteristics (Arnold et al., 2018). Pregabalin’s improvement in pain and sleep symptoms can be observed within 1–2 days of treatment, and at least 50% of patients experience sustained clinical improvement in sleep by day 11 (Arnold et al., 2015). A 2022 systematic review (Migliorini et al., 2022), which included 4,693 patients, evaluated the efficacy of different doses of pregabalin and found that 450 mg/d was most effective in improving the FIQ score, while 600 mg/d was more effective in enhancing sleep quality. However, compared to higher doses, the 300 mg/d dose had a lower incidence of adverse events, with an overall adverse event rate ranging from 70% to 90%.

Pregabalin’s therapeutic effects in FM are primarily reflected in pain relief and improved sleep. However, the incidence of adverse events is relatively high, requiring flexible dose adjustment based on patient response. The most frequently reported events include dizziness (≈1 in four participants), drowsiness (≈1 in 7), weight gain (≈1 in 18), and peripheral oedema (≈1 in 19) (Derry et al., 2016). Furthermore, there is still the drawback that only a small proportion of patients experience significant benefits from the treatment.

#### 3.2.2 Mirogabalin

Mirogabalin is an emerging calcium channel inhibitor with strong selectivity for the α2δ subunit. Compared to pregabalin, it has a slower dissociation from α2δ-1, which potentially provides longer-lasting analgesic effects (Vinik et al., 2014). This drug is primarily used for the treatment of diabetic peripheral neuropathy, and its pharmacological mechanism, similar to that of pregabalin, suggests it may have potential in treating FM (Baba et al., 2019).

Research on mirogabalin for the treatment of FM is limited, mainly focusing on animal studies. Murasawa et al. (Murasawa et al., 2020; Murasawa et al., 2021) found that mirogabalin injections significantly alleviated anxiety-like behaviors in FM model rats and improved cognitive impairment. Another animal study showed that mirogabalin significantly reduced total pain scores and increased pain thresholds in mice subjected to intermittent cold stress (Saeki et al., 2019). However, in a recent clinical trial of FM patients, neither 15 mg/d nor 30 mg/d of mirogabalin produced significant pain relief compared to placebo (Arnold et al., 2019). More trials are needed to evaluate the therapeutic effects of mirogabalin for FM.

#### 3.2.3 Gabapentin

Gabapentin is a GABA analogue, but it does not act directly on GABA receptors. Its mechanism of action is not fully understood, but it is thought to exert its analgesic effects by regulating calcium channels and reducing the release of certain neurotransmitters, such as glutamate, norepinephrine, and substance P.

In 2007, a 12-week randomized double-blind study (n = 150) found that a significantly higher proportion of FM patients taking gabapentin (1200–2400 mg/d) achieved a 30% reduction in pain compared to those taking placebo (51% vs. 31%; P = 0.014). Gabapentin also significantly improved the Brief Pain Inventory (BPI) score, the FIQ score, the PGIC, the Medical Outcomes Study Sleep Problems Index, and the MOS Short Form 36 vitality score, but did not improve the mean tender point pain threshold or the Montgomery-Asberg Depression Rating Scale (Arnold et al., 2007). Given that extended-release formulations can increase gabapentin’s bioavailability and reduce the required daily dose, potentially decreasing the frequency and severity of side effects, a 2016 15-week open-label, single-arm, single-center study enrolled 34 FM patients. Following treatment with extended-release gabapentin, pain symptoms, sleep quality, and quality of life improved, though adverse events were common (79.3%), including drowsiness, dizziness, irritability, weight gain, dry eyes, and difficulty concentrating (North et al., 2016).

Although previous studies have shown that gabapentin is effective in treating FM, there has been no significant research progress in recent years. The small number of studies available makes it insufficient to fully evaluate its benefits or potential drawbacks in FM treatment.

#### 3.2.4 Sodium oxybate

Sodium oxybate, the sodium salt of gamma-hydroxybutyrate, has an unclear mechanism of action but is known to have GABA-B receptor agonist activity. In addition to its common use for treating cataplexy and excessive daytime sleepiness associated with narcolepsy, it also has sedative effects and can improve depression and sleep quality.

Sodium oxybate has been shown to be effective in improving FM-related pain, fatigue, and sleep disturbances. In 2003, a double-blind, randomized, placebo-controlled crossover trial (Scharf et al., 2003) demonstrated that sodium oxybate significantly improved non-restorative sleep characteristics in FM patients, with good safety and tolerability. In 2009, a study involving 188 FM patients treated with 4.5 g or 6 g of sodium oxybate nightly for 8 weeks showed significant improvements in pain, sleep, and quality of life, again with good tolerability (Russell et al., 2009).

In a 2011 14-week Phase III double-blind RCT (Russell et al., 2011), sodium oxybate treatment significantly reduced pain, fatigue, and sleep disturbances, while improving functional status. The proportion of patients achieving ≥30% pain reduction was 54.2% and 58.5% in the 4.5 g and 6 g sodium oxybate groups, respectively, compared to 35.2% in the placebo group (P < 0.001). The incidence of treatment-related adverse events was also higher in the sodium oxybate groups (46.7% and 48.4%) compared to the placebo group (29.0%). Common adverse events included headache, nausea, dizziness, vomiting, diarrhea, anxiety, and sinusitis. In 2012, another Phase III trial (Spaeth et al., 2012) of sodium oxybate conducted across 108 centers in eight countries enrolled 573 FM patients, demonstrating that sodium oxybate significantly outperformed placebo in improving pain, sleep quality, and other FM-related symptoms. A 2013 prospective, multicenter, open-label extension study (Spaeth et al., 2013) lasting 38 weeks, conducted across 130 clinical sites in seven countries, further confirmed that sodium oxybate significantly improved pain VAS scores and FIQ total scores, establishing its long-term efficacy and tolerability.

## 4 Other drugs with therapeutic and developmental potential

### 4.1 Muscle relaxants

#### 4.1.1 Cyclobenzaprine

Cyclobenzaprine is a centrally acting muscle relaxant, with its mechanism of action primarily involving effects on specific regions of the brain, particularly the brainstem. By antagonizing multiple receptors, including 5-HT2A receptors, ADRA2 receptors, and H1 receptors, cyclobenzaprine reduces the excitability of motor neurons and skeletal muscles. Additionally, it helps alleviate anxiety, pain, and inflammation.

In a 2011 double-blind, randomized, placebo-controlled study (Moldofsky et al., 2011), 36 FM patients were treated with an escalating bedtime dose of ultra-low-dose cyclobenzaprine (1–4 mg) for 8 weeks. The treatment group showed significant improvements in musculoskeletal pain, fatigue, and depression compared to the placebo group. Sleep electroencephalogram analysis also indicated that the treatment group experienced an increase in restorative sleep. In a 2023 Phase III randomized controlled trial (Lederman et al., 2023) of cyclobenzaprine for FM treatment, researchers administered TNX-102 SL (a sublingual cyclobenzaprine formulation) at 2.8 mg for 2 weeks, followed by 5.6 mg or a matching placebo for 12 weeks, to 503 FM patients. TNX-102 SL treatment significantly improved pain levels, overall disease impact, and sleep quality, with good safety and tolerability. The most common adverse events were hypoesthesia in the mouth (17.3%), oral paraesthesia (5.6%), and dysgeusia (4.4%).

Compared to amitriptyline, cyclobenzaprine has similar therapeutic effects. Both drugs significantly improve symptoms with short-term treatment (1 month), but their long-term effects (6 months) are comparable to placebo (Carette et al., 1994). In terms of dosage (Santandrea et al., 1993), both a single 10 mg dose of cyclobenzaprine taken at bedtime and 10 mg three times daily significantly reduced the number of tender points, and improved sleep quality, anxiety, fatigue, irritable bowel syndrome, and stiffness in patients. However, patients receiving 30 mg/day of cyclobenzaprine (84%) reported a significantly higher frequency of side effects compared to those receiving 10 mg/day (27%), with side effects including xerostomia, drowsiness, vertigo, headache, and nervousness. In studies combining cyclobenzaprine with other medications (Cantini et al., 1994), fluoxetine (20 mg/day) combined with cyclobenzaprine (10 mg/day) showed greater improvement in pain, morning stiffness, and reduction in the number of tender points compared to cyclobenzaprine alone. Additionally, cyclobenzaprine (10 mg/day) combined with ibuprofen (600 mg) was superior to cyclobenzaprine alone (10 mg/day) only in improving morning stiffness (Fossaluzza and De Vita, 1992).

Overall, cyclobenzaprine can improve pain, fatigue, depression, and sleep quality in FM patients with good tolerability. Lower doses are associated with fewer side effects.

### 4.2 NMDA receptor modulators

#### 4.2.1 Ketamine

Ketamine, as an N-methyl-D-aspartate (NMDA) receptor antagonist, inhibits NMDA receptor channel activity, participating in the transmission of neural signals and regulation of neuronal signaling pathways. It can be used as an antidepressant treatment, as well as an anesthetic for pain relief and sedation. Graven-Nielsen et al. (2000) evaluated the effects of ketamine on FM patients. A total of 29 FM patients received an intravenous injection of either placebo or ketamine (0.3 mg/kg), and 15 patients who experienced more than a 50% reduction in pain intensity were selected for further study. The results showed that, in these 15 FM patients, ketamine significantly reduced pain VAS scores, decreased the area of local and referred pain, and increased the average pressure pain threshold at tender points (knee, epicondyle, and upper trapezius), with significant differences compared to placebo.

#### 4.2.2 Memantine hydrochloride

Memantine hydrochloride, by antagonizing NMDA receptor, modulates glutamate signaling and thus affects neurodegenerative processes, protecting neuronal cells. It is commonly used to treat moderate to severe Alzheimer’s disease. Fayed et al. (2014) treated 25 FM patients with memantine (20 mg/day) for 6 months, and the results showed that, compared to the placebo group, patients in the memantine treatment group had improved brain metabolism, with increased creatine and choline levels in the right posterior cranial muscles. A correlation between choline levels in the posterior cranial muscles and FIQ scores was also observed, along with significant improvements in measures of global impression of disease improvement and quality of life. In a study (Olivan-Blázquez et al., 2014) treating 25 FM patients with memantine hydrochloride (20 mg/day) for 6 months, it was found that compared to the placebo group, the memantine hydrochloride treatment group exhibited improved brain metabolism, with increased creatine and choline levels in the right posterior cranial muscles. A correlation was also observed between choline levels in the posterior cranial muscles and FIQ scores, along with significant improvements in measures such as PGIC and quality of life. Another RCT evaluating memantine hydrochloride treatment for FM also showed that, compared to the placebo group, 31 patients treated with memantine hydrochloride (20 mg/day) for 6 months had significant improvements in pain VAS scores, Revised Fibromyalgia Impact Questionnaire (FIQR) scores, PGIC Hospital Anxiety Depression Scale scores, and EuroQol 5D questionnaire scores. The drug was well-tolerated, with an adverse event rate of 96.7%. The most common side effects were dizziness (25.8%) and headache (12.9%) (Olivan-Blázquez et al., 2014).

#### 4.2.3 NYX-2925

NYX-2925 is a novel NMDA receptor modulator. In animal models, Ghoreishi-Haack et al. (2018) found that oral administration of NYX-2925 produced rapid and long-lasting analgesic effects in a rat model of neuropathic pain and a formalin-induced persistent pain model. Its analgesic effects were blocked by the NMDA receptor antagonist 3-(2-carboxypiperazin-4-yl)propyl-1-phosphonic acid, suggesting that NYX-2925 may have potential for treating neuropathic pain. Houck et al. (2019) further studied the safety, tolerability, and pharmacokinetics of NYX-2925 in healthy individuals. The results showed that NYX-2925 could cross the blood-brain barrier and exhibited dose-proportional pharmacokinetics, with minimal accumulation in the body after 7 days of once-daily dosing. No adverse events were observed during the study, indicating that the drug is safe and well-tolerated. The development of NYX-2925 for FM treatment has now progressed to Phase II clinical trials.

Overall, the three NMDA receptor modulators show promising therapeutic potential and warrant further research and development.

### 4.3 Cannabinoids

#### 4.3.1 Cannabidiol

Cannabidiol (CBD) is a compound extracted from the cannabis plant and belongs to the class of cannabinoids. It is one of the non-psychoactive components of the cannabis plant and, unlike tetrahydrocannabinol (THC), CBD does not affect mental state or consciousness, making it non-addictive. CBD is currently being studied for its potential to treat various conditions and symptoms, including epilepsy, anxiety, depression, pain, inflammation, and some neurological disorders. However, there are no clinical studies to date that confirm its effectiveness in treating FM (Hindley et al., 2020).

In 2021, Boehnke et al. (2021) conducted an online survey involving 878 FM patients to explore whether they used CBD as a substitute for traditional pain medications. The results showed that the majority of respondents (72.0%) reported using CBD products as a replacement for nonsteroidal anti-inflammatory drugs, opioids, gabapentinoids, and benzodiazepines. Respondents noted improvements in FM-related symptoms, including pain, fatigue, anxiety, and cognition after substituting with CBD. Additionally, those who chose to use CBD-cannabis (containing more than 0.3% Δ-9-tetrahydrocannabinol) reported greater improvements in health, pain, memory, and sleep.

#### 4.3.2 Tetrahydrocannabinol

Tetrahydrocannabinol (THC) is the primary psychoactive component of cannabis and has shown therapeutic potential for conditions such as chronic pain, insomnia, and epilepsy. However, the use of THC also comes with risks and side effects. It may impair cognitive functions, including memory, learning ability, and attention (Hindley et al., 2020).

In 2006, a study (Schley et al., 2006) summarized the effects of THC monotherapy in 9 FM patients. The daily dosage ranged from 2.5 to 15 mg, with a weekly increase of 2.5 mg. Five patients withdrew from the trial due to side effects. Although THC did not affect axon reflex erythema, daily recordings showed a significant reduction in patients’ West Haven-Yale Multidimensional Pain Inventory scores. Notably, patients who took 10–15 mg of THC experienced significant relief from electrically induced pain. In 2009, a multicenter survey study (Weber et al., 2009) provided 172 FM patients with 7.5 mg of THC daily for 7 months. Of these, 48 patients withdrew due to side effects, inadequate pain relief, or treatment costs. The remaining 124 patients experienced a significant reduction in pain intensity during THC treatment, with improvements in psychological measures, and a reduction in opioid use. Adverse events were reported by 10% of patients, primarily fatigue and dizziness, with tolerable side effects.

#### 4.3.3 Combined use of CBD and THC

In recent years, scientists have been working on cultivating cannabis strains with high CBD content and low THC content through gene editing technology to reduce its side effects in medical applications. In a 2019 randomized placebo-controlled crossover trial (van de Donk et al., 2019), the analgesic effects of a single inhalation of medicinal-grade cannabis were studied in 20 FM patients. Four different cannabis strains were tested: Bedrocan (22.4 mg THC, CBD <1 mg), Bediol (13.4 mg THC, 17.8 mg CBD), Bedrolite (18.4 mg CBD, THC <1 mg), and a placebo. The results showed that all strains had effects similar to the placebo in improving spontaneous or electrically induced pain, suggesting that a single inhalation of cannabis has limited analgesic effects for FM patients. In 2020, a Brazilian study (Chaves et al., 2020) evaluated the effects of THC-rich cannabis oil on symptoms and quality of life in FM patients. This double-blind, randomized, placebo-controlled clinical trial included 17 female FM patients over an 8-week period. The initial dosage for the cannabis oil group was one drop per day (THC 1.22 mg, CBD 0.02 mg), with gradual increases based on symptoms. The results showed a significant reduction in FIQ scores in the cannabis oil group compared to the placebo group, particularly in areas such as “feeling good”, “pain”, “work ability”, and “fatigue”. In 2021, Mazza (2021) conducted a retrospective, open-label case series study to evaluate the efficacy and adverse events of medical cannabis in the treatment of FM. The study included 38 patients who primarily used THC-dominant cannabis (200 mg/d) and a mixed THC/CBD cannabis (400 mg/d). The results showed that both short-term and long-term use of medical cannabis significantly improved patients’ pain Numerical Rating Scale scores and the Oswestry Disability Index. However, the discontinuation rate due to non-serious adverse events was high, with only 12 patients continuing treatment by the 12th month. The most common side effects were mental confusion (37%), dizziness (14%), nausea/vomiting (14%), and restlessness/irritation (14%).

In summary, research on CBD for the treatment of FM is limited, and its potential as an alternative therapeutic option requires further investigation. THC, on the other hand, has shown some therapeutic potential, with products containing higher THC levels demonstrating better efficacy. THC may offer an alternative treatment option for FM patients who do not respond to conventional therapies, but its use may be limited by adverse events. More studies are needed in the future to assess its long-term benefits.

### 4.4 Non-opioid K Ca 3.1 channel openers

#### 4.4.1 ASP0819

ASP0819 is a novel non-opioid KCa 3.1 channel opener that effectively reverses abnormal neural discharges in primary sensory afferent neurons. This drug has shown significant therapeutic potential for FM in recent years. A Phase 2a double-blind trial (Arnold et al., 2020) conducted in 2020 randomized FM patients to receive either 15 mg/day of oral ASP0819 (n = 91) or a placebo (n = 95). Results indicated that ASP0819 produced superior improvements on the Numerical Rating Scale score at weeks 2, 6, and 7 compared to the placebo; however, by week 8, there was no significant difference in pain relief between the two groups. Additional assessments demonstrated advantages in the overall impact of diseases and sleep quality. Adverse events primarily included headache, joint pain, and nausea/vomiting, with an incidence rate of 68%. Importantly, there were no major safety concerns or withdrawal effects reported. In 2021, Takeshita et al. (2021) conducted further investigations using KCa 3.1 channel-expressing cells from humans and rats, along with a rat FM model. Their findings suggest that ASP0819 activates the KCa 3.1 channel, significantly restoring muscle pressure pain thresholds and alleviating visceral and inflammatory pain by modulating nociceptive signaling in the peripheral nervous system. These results indicate the drug’s potential therapeutic efficacy for FM-like pain, warranting further development and research.

### 4.5 Iron supplementation

#### 4.5.1 Ferric carboxymaltose

Ferric carboxymaltose is an intravenous iron formulation that rapidly increases hemoglobin levels and replenishes iron stores, making it effective for treating iron deficiency anemia. Research indicates that patients with iron deficiency anemia are at a higher risk of developing FM, with serum ferritin levels below 50 ng/mL correlating with a 6.48-fold increased risk of FM (OR 6.48; 95% CI 2.03 to 20.69; P = 0.002) (Ortanci et al., 2010). Iron serves as a cofactor for enzymes involved in the synthesis of various neurotransmitters, such as serotonin and norepinephrine; thus, iron deficiency may lead to decreased levels of these substances, contributing to the development of FM.

Hamarat et al. (2023) conducted a study involving 90 female FM patients with ferritin levels below 60 mcg/dL, who received intravenous ferric carboxymaltose treatment over 4–5 weeks. Results showed a significant increase in FIQ scores compared to baseline. In another randomized, placebo-controlled, double-blind Phase 2 clinical trial (Boomershine et al., 2018), 81 iron-deficient FM patients received two intravenous injections of either ferric carboxymaltose or placebo (saline). Although the rate of improvement of ≥13 points on the FIQR score was higher in the ferric carboxymaltose group (77%) compared to the placebo group (67%), the difference was not statistically significant. However, the FIQR scores, BPI scores, fatigue VAS scores, and iron indices showed significant improvements in the ferric carboxymaltose group compared to the placebo group. Mild adverse reactions, such as flushing (14.6%), nausea (7.3%), and dizziness (4.9%), were observed, with a total adverse event rate of 29.3%, higher than the placebo group (5%). These findings suggest that FM patients should be screened for iron deficiency anemia, as appropriate iron supplementation may reduce pain and improve quality of life in those with concurrent iron deficiency anemia.

### 4.6 Endogenous cannabinoid-like substances

#### 4.6.1 Palmitoylethanolamide

Palmitoylethanolamide (PEA) is an endogenous cannabinoid-like substance classified as a nutritional amide. It is produced in the human body from N-acylethanolamines through the action of N-acyl ethanolamine hydrolase, and it interacts with cannabinoid receptors such as CB1 and CB2, helping to alleviate pain.

As early as 2012, PEA demonstrated potential for treating chronic pain. One study (Gatti et al., 2012) included 610 patients with chronic pain that was poorly controlled by conventional treatments. These patients received an additional treatment of PEA (600 mg), resulting in a significant reduction in average pain scores for all participants, regardless of the underlying condition. Importantly, no adverse effects were reported during the treatment. In animal models (Luongo et al., 2013) induced mechanical and thermal hyperalgesia in mice using formalin and found that PEA reduced both mechanical and thermal hyperalgesia in a dose-dependent manner, along with a decrease in the activation of microglia and astrocytes. A retrospective study (Schweiger et al., 2019) analyzed 359 FM patients who were treated with PEA from 2013 to 2016. The results indicated statistically significant changes over time in the patients’ pain VAS scores, quality of life scores, and FIQ scores. Only 36 patients reported gastrointestinal-related adverse reactions, such as diarrhea, dyspepsia, bloating, constipation, and vomiting.

In 2015, a study (Del Giorno et al., 2015) divided 80 FM patients into a retrospective group (45 patients) and a prospective group (35 patients). After 6 months of treatment with duloxetine and pregabalin, the retrospective group showed reductions in the number of tender points, pain frequency, and pain intensity. In contrast, the prospective group, which received duloxetine and pregabalin along with PEA for 3 months, exhibited even greater reductions in these measures compared to the retrospective group. Importantly, no adverse effects were reported in either group. A 2023 study (Salaffi et al., 2023) involving 142 FM patients, after 3 months of stable treatment with pregabalin and duloxetine, found that 62 patients who added PEA (600 mg b.i.d.) and acetyl-L-carnitine (500 mg b.i.d.) experienced improvements in the widespread pain index, FIQ scores, and modified FM Assessment Status scores.

These studies suggest that PEA may represent a new, well-tolerated treatment option for FM, particularly as an adjunct to duloxetine and pregabalin, offering additional benefits. However, high-quality randomized clinical trials are necessary to further evaluate the efficacy and safety of PEA as a standalone treatment for FM.

### 4.7 Topical hormone

#### 4.7.1 Testosterone gel

In 2015, White et al. (2015) conducted a Phase I/II prospective pilot study involving 12 female FM patients. Participants applied 0.75 g of 1% testosterone gel every morning for 28 consecutive days. The results indicated significant increases in both free testosterone levels and 24-h free testosterone levels in the patients’ serum. Additionally, participants reported notable reductions in muscle pain, stiffness, and fatigue, as well as a decrease in the number of tender points and an increase in libido. No adverse effects were observed regarding cardiovascular, hepatic, renal, or hematological functions, and no patients reported any treatment-related adverse events.

These findings suggest that while oral androgen formulations may have limited efficacy and higher side effects, topical formulations like testosterone gel show promising therapeutic potential.

### 4.8 Other medicines

#### 4.8.1 Flupirtine

Flupirtine is a non-opioid analgesic whose mechanism of action remains incompletely understood. Potential mechanisms include the enhancement of GABA activity, modulation of NMDA receptors, blockade of voltage-gated sodium channels, and reduction of norepinephrine reuptake. It exhibits analgesic, antidepressant, and sleep-improving effects without addiction or tolerance. A 2000 study (Stoll, 2000) reported successful treatment of 4 FM patients with flupirtine (average dose approximately 160 mg/day), leading to significant improvements in pain, sleep disturbances, fatigue, and depressive symptoms. However, there have been no subsequent studies on the efficacy of this drug in treating FM.

#### 4.8.2 Neurotropin

Neurotropin is a non-protein bioactive substance extracted from the inflammatory skin of rabbits inoculated with vaccinia virus. It possesses analgesic properties, although its mechanism of action remains unclear. Neurotropin has been used in Japan for over 20 years to alleviate pain associated with conditions such as low back pain and osteoarthritis, with no severe side effects reported. In an animal model experiment for FM (Nasu et al., 2019), researchers found that the depressive-like behavior in rats could be reversed by administering the antidepressant desipramine, and a single injection of neurotropin also inhibited the increase in depressive-like behavior. These findings suggest that neurotropin exhibits antidepressant effects in the FM mode.

#### 4.8.3 Gamma-hydroxybutyrate and sodium oxybate

Gamma-hydroxybutyrate (GHB) is a potent sedative with strong inhibitory effects on the central nervous system. It is colorless, odorless, and easily soluble in water, making it a potential substance for illicit use, where individuals may unknowingly ingest it, leading to symptoms such as memory loss, nausea, and vomiting. Due to this risk, GHB is strictly regulated. Sodium oxybate, which is structurally similar to GHB, may carry the same abuse potential, which could explain the limitations in its clinical application and recent research. However, there are companies currently submitting new drug applications for sodium oxybate to the U.S. FDA. If approved, sodium oxybate could offer FM patients a new treatment option.

#### 4.8.4 ASP8062

ASP8062 is a novel positive allosteric modulator of the GABA-B receptor. In 2019, researchers discovered that ASP8062 exhibits positive allosteric modulation activity on human and rat GABA-B receptors, with high efficacy, selectivity, oral bioavailability, and central nervous system penetration. In a rat model of FM, ASP8062 demonstrated analgesic effects with minimal impact on motor coordination and had positive effects on electroencephalogram activity related to sleep cycles and stages. These findings suggest that ASP8062 has the potential to be an analgesic for FM treatment, with fewer side effects compared to GABA-B receptor agonists (Murai et al., 2019).

## 5 Drugs with poor or controversial efficacy in the treatment of FM

### 5.1 Dehydroepiandrosterone

From a pathophysiological perspective, fluctuations in estrogen have been shown to increase pain intensity and lower pain thresholds, while elevated testosterone levels have been associated with increased pain thresholds. This aligns with the observation that FM is more prevalent in women, prompting research into whether androgen supplementation could alleviate FM symptoms.

Dehydroepiandrosterone (DHEA) is a precursor to synthetic hormones, and patients with FM often exhibit persistent adrenal hyporesponsiveness and low DHEA levels. In this context, Axel Finckh et al. conducted a double-blind crossover trial (Finckh et al., 2005) in which postmenopausal women with FM were randomly assigned to receive either DHEA supplementation (50 mg/day) or a placebo for 3 months, with a 1-month washout period. The results showed that median DHEA sulfate blood levels increased twofold; however, there were no improvements in symptoms such as wellbeing, pain, fatigue, cognitive function, depression, or anxiety. Furthermore, common side effects of androgen treatment—such as oily skin, acne, and increased body hair—were reported more frequently in the DHEA group compared to the placebo group.

### 5.2 Interferon-alpha

Interferon-alpha is a commonly used antiviral drug in clinical practice. In a study (Russell et al., 1999a) by Russell et al., 112 patients with FM were randomly assigned to four groups to receive sublingual interferon-alpha (15 IU, 50 IU, or 150 IU) or a placebo every morning. By the sixth week, a reduction in the CD4^+^ T cell subset expressing leukocyte antigen was observed in the 15 IU and 150 IU treatment groups. Only patients in the 50 IU group showed improvements in morning stiffness and physical function; other outcome measures did not significantly change. No adverse events related to interferon-alpha treatment were reported, indicating a favorable safety profile (Russell et al., 1999b).

### 5.3 Alpha-1 antitrypsin

Alpha-1 antitrypsin (AAT) is a protein produced in the liver and a member of the serine protease inhibitor superfamily. It serves as an important anti-inflammatory and antiprotease, primarily inhibiting the activity of proteases such as trypsin, elastase, and other proteolytic enzymes, thereby protecting body tissues from destruction by these enzymes. In 2004, Blanco et al. published a case report study (Blanco et al., 2004) involving two Spanish sisters with AAT deficiency and FM who began AAT replacement therapy with AAT infusion in 1992. Over the subsequent 6 years, their FM symptoms were controlled. However, in 1998, due to a shortage of AAT medication, they discontinued treatment for 4–6 months each year for 5 years, leading to a severe recurrence of FM symptoms. Once the medication supply returned to normal, their symptoms were alleviated again, suggesting a potential therapeutic role for AAT. However, in a 2012 randomized, double-blind, placebo-controlled, crossover study (Alegre et al., 2012), 13 FM patients completed a 9-week trial of intravenous AAT/placebo (60 mg/kg). The results showed no significant improvement in FM symptoms with AAT treatment compared to placebo.

### 5.4 Naltrexone hydrochloride

Naltrexone hydrochloride blocks the effects of opioids by competitively binding to opioid receptors in the brain and spinal cord, primarily the μ-opioid receptors, and is commonly used to treat alcohol and opioid dependence. The team led by Jarred Younger has extensively explored the use of naltrexone for FM. In 2009, Younger et al. conducted a pilot single-blind crossover trial (Younger and Mackey, 2009) assessing the efficacy of low-dose naltrexone in treating FM. 10 female participants received 2 weeks of placebo followed by 8 weeks of 4.5 mg/day naltrexone. Results showed significant symptom improvement compared to placebo, along with increased mechanical and thermal pain thresholds. Side effects, including insomnia and vivid dreams, were mild and transient.

In the same year, the team conducted a randomized double-blind crossover trial (Younger et al., 2009) involving 10 FM female patients and 10 age- and gender-matched healthy controls. However, pain threshold and tolerance changes were similar between the two groups post-treatment. In 2013, the team published a larger randomized, double-blind, placebo-controlled, crossover study (Younger et al., 2013) that included 31 FM female patients, who received 4.5 mg of naltrexone daily for 12 weeks. Compared to placebo, naltrexone significantly improved pain and overall life satisfaction, while negative emotions improved; however, fatigue and sleep issues remained unchanged. The team also investigated the mechanism of naltrexone in treating FM (Parkitny and Younger, 2017), finding that low-dose treatment significantly reduced various inflammatory markers (IL-1β, IL-1Ra, IL-2, IFN-α, TGF-α, TNF-α, etc.), suggesting that naltrexone may exert its effects by reducing key pro-inflammatory cytokines.

In 2021, Jackson et al. conducted a study (Jackson et al., 2021) at the American Addiction Centers involving 55 patients with opioid-induced hyperalgesia (OIH) and 21 FM patients. After taking low-dose naltrexone, OIH patients demonstrated over fourfold improvement in pain tolerance, while FM patients showed a twofold increase. Although the study lacked a placebo control, these results indicate that low-dose naltrexone may be effective for FM patients.

However, recent studies on low-dose naltrexone for FM treatment have yielded less promising results. In 2023, Bested et al. performed a randomized, double-blind, placebo-controlled crossover study (Bested et al., 2023) with 58 FM patients. Participants were randomly assigned to receive either 4.5 mg of naltrexone or placebo daily. The findings indicated that low-dose naltrexone did not produce clinically significant analgesic effects or improvements in physical functioning. In 2024, a single-center randomized, double-blind, placebo-controlled trial (Due Bruun et al., 2024) in Denmark investigated the efficacy of 6 mg low-dose naltrexone over 12 weeks in women with FM. 99 patients were randomized to either the low-dose naltrexone group (n = 49) or placebo group (n = 50). The results suggested that low-dose naltrexone might improve memory issues related to FM pain, but its effectiveness in pain relief did not surpass that of placebo. Recent clinical studies on naltrexone for FM treatment have employed more rigorous designs and larger sample sizes than those conducted by Jarred Younger’s team, suggesting that low-dose naltrexone may be ineffective for treating FM.

### 5.5 Tramadol

Tramadol exhibits a dual mechanism of action: it binds to μ-opioid receptors in the brain and spinal cord to provide analgesic effects, while also inhibiting the reuptake of norepinephrine and serotonin, thereby enhancing its analgesic properties. A study (Biasi et al., 1998) conducted in 1998 evaluated the analgesic effects of tramadol compared to a placebo in patients with FM. Using a double-blind crossover design, the study involved 12 patients and found that the tramadol group experienced a 20.6% reduction in pain as measured by VAS, while the placebo group reported a 19.8% increase in pain VAS scores. However, no significant clinical differences were observed in tenderness improvement between the two groups. Despite the analgesic benefits of opioids, there is insufficient evidence to support their use in treating FM. The associated risks of severe adverse events, including overdose, misuse, suicide, and death, often lead to their recommendation against in FM management.

## 6 Discussion

This review summarizes the currently clinically applied medications, those in human trials, and those tested in animals for FM treatment. Drugs that modulate serotonin or norepinephrine and affect the GABA system remain at the forefront of research. In addition to approved treatments such as pregabalin, duloxetine, and milnacipran, sodium oxybate has shown promising efficacy and research support, although its potential for abuse may limit widespread use. Other medications related to these mechanisms have demonstrated efficacy but suffer from a lack of sufficient quantity and quality of studies, providing inadequate evidence for clinical application.

Beyond these two primary mechanisms, the muscle relaxant cyclobenzaprine has reached phase III clinical trials, confirming its benefits for pain and sleep improvement. While studies on NMDA receptor modulators are limited, they show some efficacy and good tolerability. Cannabis formulations containing both CBD and THC can enhance clinical efficacy while mitigating the side effects of psychological dependence, presenting promising development potential. Iron supplements have demonstrated efficacy in FM patients with iron-deficiency anemia, suggesting potential for clinical application. Other drugs, such as palmitoylethanolamide, testosterone gel, flupirtine, and neurotropin, remain unclear in their efficacy and require further clinical investigation.

FM is a complex condition with numerous triggers and clinical manifestations. Recent years have seen advancements in understanding the pathogenesis of FM, yet consensus is still lacking. Patients often exhibit varied responses to different medications. Despite significant progress in pharmacological research over the past few decades, a cure for FM remains elusive. Currently, no drug provides consistent efficacy for the majority of FM patients, nor is there any single treatment that alleviates all symptoms. A deeper understanding of FM and its mechanisms is essential for developing more targeted therapies. Medications with preliminary evidence of efficacy are eagerly anticipated for further high-quality studies to confirm their effectiveness and safety. Compared with the current predicaments in the application of pharmacotherapy, non-pharmacological therapies such as exercise therapy (Li et al., 2025), cognitive behavioral therapy (Pardos-Gascón et al., 2021), and patient education (Antunes et al., 2022) demonstrate better efficacy and safety. Combining these non-pharmacological therapies with pharmacotherapy when treating FM may bring more benefits to patients with fibromyalgia.

## References

[B1] Ablin J. N. Amital H. Ehrenfeld M. (2013). Guidelines for the diagnosis and treatment of the fibromyalgia syndrome. Harefuah 152, 742–747. 24483001

[B2] Alegre C. Barceló M. Jardí R. Rodríguez-Frias F. Camprubí S. (2012). α1-Antitrypsin in fibromyalgia: results of a randomized, placebo-controlled, double-blind and crossover pilot trial. Musculoskelet. Care 10, 178–183. 10.1002/msc.1000 22190533

[B3] Antunes M. D. Schmitt A. C. B. Marques A. P. (2022). Amigos de fibro (fibro friends): validation of an educational program to promote health in fibromyalgia. Int. J. Environ. Res. Public Health 19, 5297. 10.3390/ijerph19095297 35564691 PMC9102409

[B4] Antunes M. D. da Rocha Loures F. C. N. de Souza I. M. B. Cruz A. T. de Oliveira Januário P. Pinheiro M. M. L. S. (2023). A web-based educational therapy intervention associated with physical exercise to promote health in fibromyalgia in Brazil: the Amigos De Fibro (Fibro Friends) study protocol. Trials 24, 655. 10.1186/s13063-023-07588-3 37814321 PMC10561409

[B5] Ariani A. Bazzichi L. Sarzi-Puttini P. Salaffi F. Manara M. Prevete I. (2021). The Italian society for rheumatology clinical practice guidelines for the diagnosis and management of fibromyalgia best practices based on current scientific evidence. Reumatismo 73, 89–105. 10.4081/reumatismo.2021.1362 34342210

[B6] Arnold L. M. Rosen A. Pritchett Y. L. D'Souza D. N. Goldstein D. J. Iyengar S. (2005). A randomized, double-blind, placebo-controlled trial of duloxetine in the treatment of women with fibromyalgia with or without major depressive disorder. Pain 119, 5–15. 10.1016/j.pain.2005.06.031 16298061

[B7] Arnold L. M. Goldenberg D. L. Stanford S. B. Lalonde J. K. Sandhu H. S. Keck P. E. Jr (2007). Gabapentin in the treatment of fibromyalgia: a randomized, double-blind, placebo-controlled, multicenter trial. Arthritis Rheum. 56, 1336–1344. 10.1002/art.22457 17393438

[B8] Arnold L. M. Clauw D. Wang F. Ahl J. Gaynor P. J. Wohlreich M. M. (2010a). Flexible dosed duloxetine in the treatment of fibromyalgia: a randomized, double-blind, placebo-controlled trial. J. Rheumatol. 37, 2578–2586. 10.3899/jrheum.100365 20843911

[B9] Arnold L. M. Chatamra K. Hirsch I. Stoker M. (2010b). Safety and efficacy of esreboxetine in patients with fibromyalgia: an 8-week, multicenter, randomized, double-blind, placebo-controlled study. Clin. Ther. 32, 1618–1632. 10.1016/j.clinthera.2010.08.003 20974319

[B10] Arnold L. M. Wang F. Ahl J. Gaynor P. J. Wohlreich M. M. (2011). Improvement in multiple dimensions of fatigue in patients with fibromyalgia treated with duloxetine: secondary analysis of a randomized, placebo-controlled trial. Arthritis Res. Ther. 13, R86. 10.1186/ar3359 21668963 PMC3218901

[B11] Arnold L. M. Hirsch I. Sanders P. Ellis A. Hughes B. (2012). Safety and efficacy of esreboxetine in patients with fibromyalgia: a fourteen-week, randomized, double-blind, placebo-controlled, multicenter clinical trial. Arthritis Rheum. 64, 2387–2397. 10.1002/art.34390 22275142

[B12] Arnold L. M. Emir B. Pauer L. Resnick M. Clair A. (2015). Time to improvement of pain and sleep quality in clinical trials of pregabalin for the treatment of fibromyalgia. Pain Med. 16, 176–185. 10.1111/pme.12636 25529830

[B13] Arnold L. M. Choy E. Clauw D. J. Oka H. Whalen E. Semel D. (2018). An evidence-based review of pregabalin for the treatment of fibromyalgia. Curr. Med. Res. Opin. 34, 1397–1409. 10.1080/03007995.2018.1450743 29519159

[B14] Arnold L. M. Whitaker S. Hsu C. Jacobs D. Merante D. (2019). Efficacy and safety of mirogabalin for the treatment of fibromyalgia: results from three 13-week randomized, double-blind, placebo- and active-controlled, parallel-group studies and a 52-week open-label extension study. Curr. Med. Res. Opin. 35, 1825–1835. 10.1080/03007995.2019.1629757 31284771

[B15] Arnold L. M. Blauwet M. B. Tracy K. Cai N. Walzer M. Blahunka P. (2020). Efficacy and safety of ASP0819 in patients with fibromyalgia: results of a proof-of-concept, randomized, double-blind, placebo-controlled trial. J. Pain Res. 13, 3355–3369. 10.2147/JPR.S274562 33328761 PMC7735791

[B16] Baba M. Matsui N. Kuroha M. Wasaki Y. Ohwada S. (2019). Mirogabalin for the treatment of diabetic peripheral neuropathic pain: a randomized, double-blind, placebo-controlled phase III study in Asian patients. J. Diabetes Investig. 10, 1299–1306. 10.1111/jdi.13013 30672128 PMC6717827

[B17] Banerjee S. High J. Stirling S. Shepstone L. Swart A. M. Telling T. (2021). Study of mirtazapine for agitated behaviours in dementia (SYMBAD): a randomised, double-blind, placebo-controlled trial. Lancet 398, 1487–1497. 10.1016/S0140-6736(21)01210-1 34688369 PMC8546216

[B18] Bartley E. J. Fillingim R. B. (2013). Sex differences in pain: a brief review of clinical and experimental findings. Br. J. Anaesth. 111, 52–58. 10.1093/bja/aet127 23794645 PMC3690315

[B19] Bested K. Jensen L. M. Andresen T. Tarp G. Skovbjerg L. Johansen T. S. D. (2023). Low-dose naltrexone for treatment of pain in patients with fibromyalgia: a randomized, double-blind, placebo-controlled, crossover study. Pain Rep. 8, e1080. 10.1097/PR9.0000000000001080 38226027 PMC10789452

[B20] Biasi G. Manca S. Manganelli S. Marcolongo R. (1998). Tramadol in the fibromyalgia syndrome: a controlled clinical trial *versus* placebo. Int. J. Clin. Pharmacol. Res. 18, 13–19. 9604730

[B21] Blanco I. Canto H. de Serres F. J. Fernandez-Bustillo E. Rodríguez M. C. (2004). Alpha1-antitrypsin replacement therapy controls fibromyalgia symptoms in 2 patients with PI ZZ alpha1-antitrypsin deficiency. J. Rheumatol. 31, 2082–2085. 15468381

[B22] Boehnke K. F. Gagnier J. J. Matallana L. Williams D. A. (2021). Substituting cannabidiol for opioids and pain medications among individuals with fibromyalgia: a large online survey. J. Pain 22, 1418–1428. 10.1016/j.jpain.2021.04.011 33992787 PMC8578153

[B23] Boomershine C. S. Koch T. A. Morris D. (2018). A blinded, randomized, placebo-controlled study to investigate the efficacy and safety of ferric carboxymaltose in iron-deficient patients with fibromyalgia. Rheumatol. Ther. 5, 271–281. 10.1007/s40744-017-0088-9 29149437 PMC5935608

[B24] Cantini F. Bellandi F. Niccoli L. Di Munno O. (1994). Fluoxetin combined with cyclobenzaprine in the treatment of fibromyalgia. Minerva Med. 85, 97–100. 8196850

[B25] Carette S. Bell M. J. Reynolds W. J. Haraoui B. McCain G. A. Bykerk V. P. (1994). Comparison of amitriptyline, cyclobenzaprine, and placebo in the treatment of fibromyalgia. A randomized, double-blind clinical trial. Arthritis Rheum. 37, 32–40. 10.1002/art.1780370106 8129762

[B26] Chappell A. S. Littlejohn G. Kajdasz D. K. Scheinberg M. D'Souza D. N. Moldofsky H. (2009). A 1-year safety and efficacy study of duloxetine in patients with fibromyalgia. Clin. J. Pain 25, 365–375. 10.1097/AJP.0b013e31819be587 19454869

[B27] Chaves C. Bittencourt P. C. T. Pelegrini A. (2020). Ingestion of a THC-Rich cannabis oil in people with fibromyalgia: a randomized, double-blind, placebo-controlled clinical trial. Pain Med. 21, 2212–2218. 10.1093/pm/pnaa303 33118602 PMC7593796

[B28] China Association of Chinese Medicine Rheumatology Society, Fibromyalgia Research Group of Traditional Chinese and Western Medicine in Rheumatology and Immunology, Capital Institute of Rheumatology and Immunology of Integrated Chinese and Western Medicine. (2023). Chinese recommendations for the management of fibromyalgia syndrome. Zhonghua Nei Ke Za Zhi. 62 (2), 129–146. 10.3760/cma.j.cn112138-20220602-00426 36740404

[B29] Clauw D. J. (2014). Fibromyalgia: a clinical review. JAMA 311, 1547–1555. 10.1001/jama.2014.3266 24737367

[B30] Cording M. Derry S. Phillips T. Moore R. A. Wiffen P. J. (2015). Milnacipran for pain in fibromyalgia in adults. Cochrane Database Syst. Rev. 2015, CD008244. 10.1002/14651858.CD008244.pub3 26482422 PMC6481368

[B31] Del Giorno R. Skaper S. Paladini A. Varrassi G. Coaccioli S. (2015). Palmitoylethanolamide in fibromyalgia: results from prospective and retrospective observational studies. Pain Ther. 4, 169–178. 10.1007/s40122-015-0038-6 26334329 PMC4676767

[B32] Derry S. Cording M. Wiffen P. J. Law S. Phillips T. Moore R. A. (2016). Pregabalin for pain in fibromyalgia in adults. Cochrane Database Syst. Rev. 9, CD011790. 10.1002/14651858.CD011790.pub2 27684492 PMC6457745

[B33] Díaz-Marsá M. Palomares N. Morón M. D. Tajima K. Fuentes M. E. López-Ibor J. J. (2011). Psychological factors affecting response to antidepressant drugs in fibromyalgia. Psychosomatics 52, 237–244. 10.1016/j.psym.2010.12.014 21565595

[B34] Due Bruun K. Christensen R. Amris K. Vaegter H. B. Blichfeldt-Eckhardt M. R. Bye-Møller L. (2024). Naltrexone 6 mg once daily *versus* placebo in women with fibromyalgia: a randomised, double-blind, placebo-controlled trial. Lancet Rheumatol. 6, e31–e39. 10.1016/S2665-9913(23)00278-3 38258677

[B35] Dwight M. M. Arnold L. M. O’Brien H. Metzger R. Morris-Park E. Keck P. E. Jr (1998). An open clinical trial of venlafaxine treatment of fibromyalgia. Psychosomatics 39, 14–17. 10.1016/S0033-3182(98)71375-1 9538670

[B36] D’Onghia M. Ciaffi J. Ruscitti P. Cipriani P. Giacomelli R. Ablin J. N. (2022). The economic burden of fibromyalgia: a systematic literature review. Semin. Arthritis Rheum. 56, 152060. 10.1016/j.semarthrit.2022.152060 35849890

[B37] Farag H. M. Yunusa I. Goswami H. Sultan I. Doucette J. A. Eguale T. (2022). Comparison of amitriptyline and US food and drug administration-approved treatments for fibromyalgia: a systematic review and network meta-analysis. JAMA Netw. Open 5, e2212939. 10.1001/jamanetworkopen.2022.12939 35587348 PMC9121190

[B38] Fayed N. Olivan-Blázquez B. Herrera-Mercadal P. Puebla-Guedea M. Pérez-Yus M. C. Andrés E. (2014). Changes in metabolites after treatment with memantine in fibromyalgia. A double-blind randomized controlled trial with magnetic resonance spectroscopy with a 6-month follow-up. CNS Neurosci. Ther. 20, 999–1007. 10.1111/cns.12314 25230216 PMC6493189

[B39] Fillingim R. B. King C. D. Ribeiro-Dasilva M. C. Rahim-Williams B. Riley J. L. 3rd (2009). Sex, gender, and pain: a review of recent clinical and experimental findings. J. Pain 10, 447–485. 10.1016/j.jpain.2008.12.001 19411059 PMC2677686

[B40] Finckh A. Berner I. C. Aubry-Rozier B. So A. K. L. (2005). A randomized controlled trial of dehydroepiandrosterone in postmenopausal women with fibromyalgia. J. Rheumatol. 32, 1336–1340. 15996074

[B41] Fitzcharles M.-A. Ste-Marie P. A. Goldenberg D. L. (2012). Canadian guidelines for the diagnosis and management of fibromyalgia syndrome: executive summary. Pain Res. Manag. 18, 918216. 10.1155/2013/918216 PMC3673928 23748251

[B42] Fossaluzza V. De Vita S. (1992). Combined therapy with cyclobenzaprine and ibuprofen in primary fibromyalgia syndrome. Int. J. Clin. Pharmacol. Res. 12, 99–102. 1428304

[B43] Gatti A. Lazzari M. Gianfelice V. Di Paolo A. Sabato E. Sabato A. F. (2012). Palmitoylethanolamide in the treatment of chronic pain caused by different etiopathogenesis. Pain Med. 13, 1121–1130. 10.1111/j.1526-4637.2012.01432.x 22845893

[B44] Ghoreishi-Haack N. Priebe J. M. Aguado J. D. Colechio E. M. Burgdorf J. S. Bowers M. S. (2018). NYX-2925 is a novel N-Methyl-d-Aspartate receptor modulator that induces rapid and long-lasting analgesia in rat models of neuropathic pain. J. Pharmacol. Exp. Ther. 366, 485–497. 10.1124/jpet.118.249409 29986951

[B45] Giorgi V. Bazzichi L. Batticciotto A. Pellegrino G. Di Franco M. Sirotti S. (2023). Fibromyalgia: one year in review 2023. Clin. Exp. Rheumatol. 41, 1205–1213. 10.55563/clinexprheumatol/257e99 37378487

[B46] Graven-Nielsen T. Kendall S. A. Henriksson K. G. Bengtsson M. Sörensen J. Johnson A. (2000). Ketamine reduces muscle pain, temporal summation, and referred pain in fibromyalgia patients. Pain 85, 483–491. 10.1016/S0304-3959(99)00308-5 10781923

[B47] Gupta H. Girma B. Jenkins J. S. Kaufman S. E. Lee C. A. Kaye A. D. (2021). Milnacipran for the treatment of fibromyalgia. Health Psychol. Res. 9, 25532. 10.52965/001c.25532 34746490 PMC8567778

[B48] Hamarat H. Gürcü S. Kıvanç B. K. Aydemir A. E. (2023). Ferric carboxymaltose therapy reduces pain and improves the quality of life in female patients with fibromyalgia. Eur. Rev. Med. Pharmacol. Sci. 27, 10375–10380. 10.26355/eurrev_202311_34311 37975360

[B49] Häuser W. Thieme K. Turk D. C. (2010). Guidelines on the management of fibromyalgia syndrome – a systematic review. Eur. J. Pain 14, 5–10. 10.1016/j.ejpain.2009.01.006 19264521

[B50] Häuser W. Petzke F. Üçeyler N. Sommer C. (2011). Comparative efficacy and acceptability of amitriptyline, duloxetine and milnacipran in fibromyalgia syndrome: a systematic review with meta-analysis. Rheumatology 50, 532–543. 10.1093/rheumatology/keq354 21078630

[B51] Häuser W. Urrútia G. Tort S. Uçeyler N. Walitt B. (2013). Serotonin and noradrenaline reuptake inhibitors (SNRIs) for fibromyalgia syndrome. Cochrane Db Syst. Rev., CD010292. 10.1002/14651858.CD010292 23440848

[B52] Hindley G. Beck K. Borgan F. Ginestet C. E. McCutcheon R. Kleinloog D. (2020). Psychiatric symptoms caused by cannabis constituents: a systematic review and meta-analysis. Lancet Psychiatry 7, 344–353. 10.1016/S2215-0366(20)30074-2 32197092 PMC7738353

[B53] Houck D. R. Sindelar L. Sanabria C. R. Stanworth S. H. Krueger M. Suh M. (2019). NYX-2925, a novel N-methyl-D-aspartate receptor modulator: a first-in-human, randomized, double-blind study of safety and pharmacokinetics in adults. Clin. Transl. Sci. 12, 164–171. 10.1111/cts.12584 30242962 PMC6440576

[B54] Jackson D. Singh S. Zhang-James Y. Faraone S. Johnson B. (2021). The effects of low dose naltrexone on opioid induced hyperalgesia and fibromyalgia. Front. Psychiatry 12, 593842. 10.3389/fpsyt.2021.593842 33664680 PMC7921161

[B55] Kleinman N. L. Sanchez R. J. Lynch W. D. Cappelleri J. C. Beren I. A. Joshi A. V. (2011). Health outcomes and costs among employees with fibromyalgia treated with pregabalin vs. standard of care. Pain Pract. 11, 540–551. 10.1111/j.1533-2500.2011.00453.x 21392253

[B56] Lederman S. Arnold L. M. Vaughn B. Kelley M. Sullivan G. M. (2023). Efficacy and safety of sublingual cyclobenzaprine for the treatment of fibromyalgia: results from a randomized, double-blind, placebo-controlled trial. Arthritis Care Res. Hob. 75, 2359–2368. 10.1002/acr.25142 37165930

[B57] Lenert M. E. Avona A. Garner K. M. Barron L. R. Burton M. D. (2021). Sensory neurons, neuroimmunity, and pain modulation by sex hormones. Endocrinology 162, bqab109. 10.1210/endocr/bqab109 34049389 PMC8237991

[B58] Li Y. Long M. Li Y. Jing B. Wang Y. Li Z. (2025). Comparative efficacy of ba-duan-jin and pregabalin in the treatment of fibromyalgia: an RCT with an exploratory analysis based on fMRI. J. Pain 34, 105487. 10.1016/j.jpain.2025.105487 40615016

[B59] Lian Y.-N. Wang Y. Zhang Y. Yang C. X. (2020). Duloxetine for pain in fibromyalgia in adults: a systematic review and a meta-analysis. Int. J. Neurosci. 130, 71–82. 10.1080/00207454.2019.1664510 31487217

[B60] Liu Y. Qian C. Yang M. (2016). Treatment patterns associated with ACR-Recommended medications in the management of fibromyalgia in the United States. J. Manag. Care Spec. Pharm. 22, 263–271. 10.18553/jmcp.2016.22.3.263 27003556 PMC10398128

[B61] Liu M. Harris S. Andreou A. P. Bo X. Al-Kaisy A. (2025). Gender differences in clinical presentations and sensory profiles in patients with fibromyalgia: implications of peripheral and central mechanisms. PAIN Rep. 10, e1229. 10.1097/PR9.0000000000001229 39816906 PMC11732657

[B62] Lunn M. P. T. Hughes R. A. C. Wiffen P. J. (2014). Duloxetine for treating painful neuropathy, chronic pain or fibromyalgia. Cochrane Database Syst. Rev. 2014, CD007115. 10.1002/14651858.CD007115.pub3 24385423 PMC10711341

[B63] Luongo L. Guida F. Boccella S. Bellini G. Gatta L. Rossi F. (2013). Palmitoylethanolamide reduces formalin-induced neuropathic-like behaviour through spinal glial/microglial phenotypical changes in mice. CNS Neurol. Disord. Drug Targets 12, 45–54. 10.2174/1871527311312010009 23394524

[B64] Macfarlane G. J. Kronisch C. Dean L. E. Atzeni F. Häuser W. Fluß E. (2017). EULAR revised recommendations for the management of fibromyalgia. Ann. Rheum. Dis. 76, 318–328. 10.1136/annrheumdis-2016-209724 27377815

[B65] Mazza M. (2021). Medical cannabis for the treatment of fibromyalgia syndrome: a retrospective, open-label case series. J. Cannabis Res. 3, 4. 10.1186/s42238-021-00060-6 33597032 PMC7890993

[B66] Mehta P. Basu A. Ahmed S. (2022). Effectiveness and adverse effects of the use of mirtazapine as compared to duloxetine for fibromyalgia: Real-life data from a retrospective cohort. Rheumatol. Int. 42, 1549–1554. 10.1007/s00296-022-05135-y 35475940

[B67] Migliorini F. Maffulli N. Knobe M. Tenze G. Aljalloud A. Colarossi G. (2022). Pregabalin administration in patients with fibromyalgia: a Bayesian network meta-analysis. Sci. Rep. 12, 12148. 10.1038/s41598-022-16146-x 35840702 PMC9287452

[B68] Migliorini F. Maffulli N. Eschweiler J. Baroncini A. Bell A. Colarossi G. (2023). Duloxetine for fibromyalgia syndrome: a systematic review and meta-analysis. J. Orthop. Surg. Res. 18, 504. 10.1186/s13018-023-03995-z 37461044 PMC10351165

[B69] Miki K. Murakami M. Oka H. Onozawa K. Yoshida S. Osada K. (2016). Efficacy of mirtazapine for the treatment of fibromyalgia without concomitant depression: a randomized, double-blind, placebo-controlled phase IIa study in Japan. Pain 157, 2089–2096. 10.1097/j.pain.0000000000000622 27218868 PMC4988084

[B70] Mohs R. Mease P. Arnold L. M. Wang F. Ahl J. Gaynor P. J. (2012). The effect of duloxetine treatment on cognition in patients with fibromyalgia. Psychosom. Med. 74, 628–634. 10.1097/PSY.0b013e31825b9855 22753629

[B71] Moldofsky H. Harris H. W. Archambault W. T. Kwong T. Lederman S. (2011). Effects of bedtime very low dose cyclobenzaprine on symptoms and sleep physiology in patients with fibromyalgia syndrome: a double-blind randomized placebo-controlled study. J. Rheumatol. 38, 2653–2663. 10.3899/jrheum.110194 21885490

[B72] Moore R. A. Derry S. Aldington D. Cole P. Wiffen P. J. (2019). Amitriptyline for fibromyalgia in adults. Cochrane Db Syst. Rev. 5, CD011824. 10.1002/14651858.CD011824 35658166 PMC6485478

[B73] Moshrif A. Shoaeir M. Z. Abbas A. S. Abdel-Aziz T. M. Gouda W. (2022). Evaluating gender differences in egyptian fibromyalgia patients using the 1990, 2011, and 2016 ACR criteria. Open Access Rheumatol. 14, 67–74. 10.2147/OARRR.S358255 35492891 PMC9046688

[B74] Murai N. Kondo Y. Akuzawa S. Mihara T. Shiraishi N. Kakimoto S. (2019). A novel GABAB receptor positive allosteric modulator, ASP8062, exerts analgesic effects in a rat model of fibromyalgia. Eur. J. Pharmacol. 865, 172750. 10.1016/j.ejphar.2019.172750 31647906

[B75] Murasawa H. Kobayashi H. Yasuda S.-I. Saeki K. Domon Y. Arakawa N. (2020). Anxiolytic-like effects of mirogabalin, a novel ligand for α2δ ligand of voltage-gated calcium channels, in rats repeatedly injected with acidic saline intramuscularly, as an experimental model of fibromyalgia. Pharmacol. Rep. 72, 571–579. 10.1007/s43440-020-00103-4 32270470

[B76] Murasawa H. Pawlak A. Kobayashi H. Saeki K. Yasuda S. I. Kitano Y. (2021). Mirogabalin, a novel ligand for α2δ subunit of voltage-gated calcium channels, improves cognitive impairments in repeated intramuscular acidic saline injection model rats, an experimental model of fibromyalgia. Biomed. Pharmacother. 139, 111647. 10.1016/j.biopha.2021.111647 33940507

[B77] Nasu T. Kubo A. Queme L. F. Mizumura K. (2019). A single administration of Neurotropin reduced the elongated immobility time in the forced swimming test of rats exposed to repeated cold stress. Behav. Pharmacol. 30, 547–554. 10.1097/FBP.0000000000000488 31188139

[B78] Neyama H. Dozono N. Uchida H. Ueda H. (2020). Mirtazapine, an α2 antagonist-type antidepressant, reverses pain and lack of morphine analgesia in fibromyalgia-like mouse models. J. Pharmacol. Exp. Ther. 375, 1–9. 10.1124/jpet.120.265942 32665319

[B79] North J. M. Hong K.-S. J. Rauck R. L. (2016). The effect of a novel form of extended-release Gabapentin on pain and sleep in fibromyalgia subjects: an open-label pilot study. Pain Pract. 16, 720–729. 10.1111/papr.12319 26059271

[B80] Olivan-Blázquez B. Herrera-Mercadal P. Puebla-Guedea M. Pérez-Yus M. C. Andrés E. Fayed N. (2014). Efficacy of memantine in the treatment of fibromyalgia: a double-blind, randomised, controlled trial with 6-month follow-up. PAIN ®. 155 (12), 2517–2525. 10.1016/j.pain.2014.09.004 25218600

[B81] Ortancil O. Sanli A. Eryuksel R. Basaran A. Ankarali H. (2010). Association between serum ferritin level and fibromyalgia syndrome. Eur. J. Clin. Nutr. 64, 308–312. 10.1038/ejcn.2009.149 20087382

[B82] Pardos-Gascón E. M. Narambuena L. Leal-Costa C. van-der Hofstadt-Román C. J. (2021). Differential efficacy between cognitive-behavioral therapy and mindfulness-based therapies for chronic pain: systematic review. Int. J. Clin. Health Psychol. 21, 100197. 10.1016/j.ijchp.2020.08.001 33363580 PMC7753033

[B83] Parkitny L. Younger J. (2017). Reduced pro-inflammatory cytokines after eight weeks of low-dose naltrexone for fibromyalgia. Biomedicines 5, 16. 10.3390/biomedicines5020016 28536359 PMC5489802

[B84] Patkar A. A. Masand P. S. Krulewicz S. Mannelli P. Peindl K. Beebe K. L. (2007). A randomized, controlled, trial of controlled release paroxetine in fibromyalgia. Am. J. Med. 120, 448–454. 10.1016/j.amjmed.2006.06.006 17466657

[B85] Pauer L. Winkelmann A. Arsenault P. Jespersen A. Whelan L. Atkinson G. (2011). An international, randomized, double-blind, placebo-controlled, phase III trial of pregabalin monotherapy in treatment of patients with fibromyalgia. J. Rheumatol. 38, 2643–2652. 10.3899/jrheum.110569 21965636

[B86] Rodrigues-Amorim D. Olivares J. M. Spuch C. Rivera-Baltanás T. (2020). A systematic review of efficacy, safety, and tolerability of duloxetine. Front. Psychiatry 11, 554899. 10.3389/fpsyt.2020.554899 33192668 PMC7644852

[B87] Russell I. J. Vipraio G. A. Michalek J. E. Craig F. E. Kang Y. K. Richards A. B. (1999a). Lymphocyte markers and natural killer cell activity in fibromyalgia syndrome: effects of low-dose, sublingual use of human interferon-alpha. J. Interferon Cytokine Res. 19, 969–978. 10.1089/107999099313523 10476945

[B88] Russell I. J. Michalek J. E. Kang Y. K. Richards A. B. (1999b). Reduction of morning stiffness and improvement in physical function in fibromyalgia syndrome patients treated sublingually with low doses of human interferon-alpha. J. Interferon Cytokine Res. 19, 961–968. 10.1089/107999099313514 10476944

[B89] Russell I. J. Perkins A. T. Michalek J. E. (2009). Sodium oxybate relieves pain and improves function in fibromyalgia syndrome: a randomized, double‐blind, placebo‐controlled, multicenter clinical trial. Arthritis and Rheumatism. 60, 299–309. 10.1002/art.24142 19116896

[B90] Russell J. I. Holman A. J. Swick T. J. Alvarez-Horine S. Wang G. Y. Guinta D. (2011). Sodium oxybate reduces pain, fatigue, and sleep disturbance and improves functionality in fibromyalgia: results from a 14-week, randomized, double-blind, placebo-controlled study. Pain 152, 1007–1017. 10.1016/j.pain.2010.12.022 21397402

[B91] Santandrea S. Montrone F. Sarzi-Puttini P. Boccassini L. Caruso I. (1993). A double-blind crossover study of two cyclobenzaprine regimens in primary fibromyalgia syndrome. J. Int. Med. Res. 21, 74–80. 10.1177/030006059302100202 8243792

[B92] Saeki K. Yasuda S.-I. Kato M. Kano M. Domon Y. Arakawa N. (2019). Analgesic effects of mirogabalin, a novel ligand for α2δ subunit of voltage-gated calcium channels, in experimental animal models of fibromyalgia. Naunyn Schmiedeb. Arch. Pharmacol. 392, 723–728. 10.1007/s00210-019-01628-z 30770951

[B93] Salaffi F. Farah S. Sarzi-Puttini P. Di Carlo M. (2023). Palmitoylethanolamide and acetyl-L-carnitine act synergistically with duloxetine and pregabalin in fibromyalgia: results of a randomised controlled study. Clin. Exp. Rheumatol. 41, 1323–1331. 10.55563/clinexprheumatol/pmdzcq 37378482

[B94] Sarzi-Puttini P. Giorgi V. Marotto D. Atzeni F. (2020). Fibromyalgia: an update on clinical characteristics, aetiopathogenesis and treatment. Nat. Rev. Rheumatol. 16, 645–660. 10.1038/s41584-020-00506-w 33024295

[B95] Sayar K. Aksu G. Ak I. Tosun M. (2003). Venlafaxine treatment of fibromyalgia. Ann. Pharmacother. 37, 1561–1565. 10.1345/aph.1D112 14565792

[B96] Schaefer C. Mann R. Masters E. T. Cappelleri J. C. Daniel S. R. Zlateva G. (2016). The comparative burden of chronic widespread pain and fibromyalgia in the United States. Pain Pract. 16, 565–579. 10.1111/papr.12302 25980433

[B97] Scharf M. B. Baumann M. Berkowitz D. V. (2003). The effects of sodium oxybate on clinical symptoms and sleep patterns in patients with fibromyalgia. J. Rheumatol. 30, 1070–1074. 12734908

[B98] Schley M. Legler A. Skopp G. Schmelz M. Konrad C. Rukwied R. (2006). Delta-9-THC based monotherapy in fibromyalgia patients on experimentally induced pain, axon reflex flare, and pain relief. Curr. Med. Res. Opin. 22, 1269–1276. 10.1185/030079906x112651 16834825

[B99] Schweiger V. Martini A. Bellamoli P. Donadello K. Schievano C. Balzo G. D. (2019). Ultramicronized palmitoylethanolamide (um-PEA) as Add-on treatment in fibromyalgia syndrome (FMS): retrospective observational study on 407 patients. CNS Neurol. Disord. Drug Targets 18, 326–333. 10.2174/1871527318666190227205359 30827269

[B100] Sencan S. Ak S. Karan A. Muslumanoglu L. Ozcan E. Berker E. (2004). A study to compare the therapeutic efficacy of aerobic exercise and paroxetine in fibromyalgia syndrome. J. Back Musculoskelet. Rehabil. 17, 57–61. 10.3233/BMR-2004-17204

[B101] Siracusa R. Paola R. D. Cuzzocrea S. Impellizzeri D. (2021). Fibromyalgia: pathogenesis, mechanisms, diagnosis and treatment options update. Int. J. Mol. Sci. 22, 3891. 10.3390/ijms22083891 33918736 PMC8068842

[B102] Spaeth M. Bennett R. M. Benson B. A. Wang Y. G. Lai C. Choy E. H. (2012). Sodium oxybate therapy provides multidimensional improvement in fibromyalgia: results of an international phase 3 trial. Ann. Rheum. Dis. 71, 935–942. 10.1136/annrheumdis-2011-200418 22294641 PMC3371223

[B103] Spaeth M. Alegre C. Perrot S. Wang Y. Guinta D. R. Alvarez-Horine S. (2013). Long-term tolerability and maintenance of therapeutic response to sodium oxybate in an open-label extension study in patients with fibromyalgia. Arthritis Res. Ther. 15, R185. 10.1186/ar4375 24286114 PMC3978755

[B104] Stoll A. L. (2000). Fibromyalgia symptoms relieved by flupirtine: an open-label case series. Psychosomatics 41, 371–372. 10.1176/appi.psy.41.4.371 10906366

[B105] Takeshita N. Oe T. Kiso T. Kakimoto S. (2021). A KCa3.1 channel opener, ASP0819, modulates nociceptive signal processing from peripheral nerves in fibromyalgia-like pain in rats. J. Pain Res. 14, 23–34. 10.2147/JPR.S274563 33469353 PMC7811477

[B106] Taylor C. P. Angelotti T. Fauman E. (2007). Pharmacology and mechanism of action of pregabalin: the calcium channel alpha2-delta (Alpha2-delta) subunit as a target for antiepileptic drug discovery. Epilepsy Res. 73, 137–150. 10.1016/j.eplepsyres.2006.09.008 17126531

[B107] Üçeyler N. Sommer C. Walitt B. Häuser W. (2017). WITHDRAWN: anticonvulsants for fibromyalgia. Cochrane Database Syst. Rev. 10, CD010782. 10.1002/14651858.CD010782.pub2 28991361 PMC6485337

[B108] van de Donk T. Niesters M. Kowal M. A. Olofsen E. Dahan A. van Velzen M. (2019). An experimental randomized study on the analgesic effects of pharmaceutical-grade cannabis in chronic pain patients with fibromyalgia. Pain 160, 860–869. 10.1097/j.pain.0000000000001464 30585986 PMC6430597

[B109] VanderWeide L. A. Smith S. M. Trinkley K. E. (2015). A systematic review of the efficacy of venlafaxine for the treatment of fibromyalgia. J. Clin. Pharm. Ther. 40, 1–6. 10.1111/jcpt.12216 25294655

[B110] Vinik A. Rosenstock J. Sharma U. Feins K. Hsu C. Merante D. (2014). Efficacy and safety of mirogabalin (DS-5565) for the treatment of diabetic peripheral neuropathic pain: a randomized, double-blind, placebo- and active comparator-controlled, adaptive proof-of-concept phase 2 study. Diabetes Care 37, 3253–3261. 10.2337/dc14-1044 25231896

[B111] Weber J. Schley M. Casutt M. Gerber H. Schuepfer G. Rukwied R. (2009). Tetrahydrocannabinol (delta 9-THC) treatment in chronic central neuropathic pain and fibromyalgia patients: results of a multicenter survey. Anesthesiol. Res. Pract. 2009, 827290. 10.1155/2009/827290 20798872 PMC2925209

[B112] White H. D. Brown L. A. J. Gyurik R. J. Manganiello P. D. Robinson T. D. Hallock L. S. (2015). Treatment of pain in fibromyalgia patients with testosterone gel: pharmacokinetics and clinical response. Int. Immunopharmacol. 27, 249–256. 10.1016/j.intimp.2015.05.016 26004317

[B113] Wolfe F. Smythe H. A. Yunus M. B. Bennett R. M. Bombardier C. Goldenberg D. L. (1990). The American college of rheumatology 1990 criteria for the classification of fibromyalgia. Report of the multicenter criteria committee. Arthritis Rheum. 33, 160–172. 10.1002/art.1780330203 2306288

[B114] Yeephu S. Suthisisang C. Suttiruksa S. Prateepavanich P. Limampai P. Russell I. J. (2013). Efficacy and safety of mirtazapine in fibromyalgia syndrome patients: a randomized placebo-controlled pilot study. Ann. Pharmacother. 47 (7-8), 921–932. 10.1345/aph.1R725 23737510

[B115] Younger J. Mackey S. (2009). Fibromyalgia symptoms are reduced by low-dose naltrexone: a pilot study. Pain Med. 10, 663–672. 10.1111/j.1526-4637.2009.00613.x 19453963 PMC2891387

[B116] Younger J. W. Zautra A. J. Cummins E. T. (2009). Effects of naltrexone on pain sensitivity and mood in fibromyalgia: no evidence for endogenous opioid pathophysiology. PLoS One 4, e5180. 10.1371/journal.pone.0005180 19365548 PMC2664472

[B117] Younger J. Noor N. McCue R. Mackey S. (2013). Low-dose naltrexone for the treatment of fibromyalgia: findings of a small, randomized, double-blind, placebo-controlled, counterbalanced, crossover trial assessing daily pain levels. Arthritis Rheum. 65, 529–538. 10.1002/art.37734 23359310

